# Sponge Microbiota Are a Reservoir of Functional Antibiotic Resistance Genes

**DOI:** 10.3389/fmicb.2016.01848

**Published:** 2016-11-17

**Authors:** Dennis Versluis, Mari Rodriguez de Evgrafov, Morten O. A. Sommer, Detmer Sipkema, Hauke Smidt, Mark W. J. van Passel

**Affiliations:** ^1^Laboratory of Microbiology, Wageningen UniversityWageningen, Netherlands; ^2^Novo Nordisk Foundation Center for Biosustainability, Technical University of DenmarkHørsholm, Denmark; ^3^National Institute for Public Health and the EnvironmentBilthoven, Netherlands

**Keywords:** antibiotic resistance, sponge, microbiota, resistance gene, functional metagenomics

## Abstract

Wide application of antibiotics has contributed to the evolution of multi-drug resistant human pathogens, resulting in poorer treatment outcomes for infections. In the marine environment, seawater samples have been investigated as a resistance reservoir; however, no studies have methodically examined sponges as a reservoir of antibiotic resistance. Sponges could be important in this respect because they often contain diverse microbial communities that have the capacity to produce bioactive metabolites. Here, we applied functional metagenomics to study the presence and diversity of functional resistance genes in the sponges *Aplysina aerophoba, Petrosia ficiformis*, and *Corticium candelabrum*. We obtained 37 insert sequences facilitating resistance to D-cycloserine (*n* = 6), gentamicin (*n* = 1), amikacin (*n* = 7), trimethoprim (*n* = 17), chloramphenicol (*n* = 1), rifampicin (*n* = 2) and ampicillin (*n* = 3). Fifteen of 37 inserts harbored resistance genes that shared <90% amino acid identity with known gene products, whereas on 13 inserts no resistance gene could be identified with high confidence, in which case we predicted resistance to be mainly mediated by antibiotic efflux. One marine-specific ampicillin-resistance-conferring β-lactamase was identified in the genus *Pseudovibrio* with 41% global amino acid identity to the closest β-lactamase with demonstrated functionality, and subsequently classified into a new family termed PSV. Taken together, our results show that sponge microbiota host diverse and novel resistance genes that may be harnessed by phylogenetically distinct bacteria.

## Introduction

In the last decades the massive medical and veterinary use of antibiotics has contributed to the selection of multi-drug resistant human pathogens, resulting in poorer treatment outcomes upon infection (Arias and Murray, 2009). Bacterial pathogens can evolve resistance vertically (Lenski, 1998) but mainly acquire resistance through horizontal gene transfer (Högberg et al., 2010), where resistance genes are obtained either from the indigenous human microbiota or from environmental microorganisms to which an individual is exposed (e.g., via food, water or soil). The current complement of resistance genes encountered in the human microbiota is suggested to have originated from natural environments, likely in part selected by prior anthropogenic antibiotic pressure (Teuber et al., 1999; Davies and Davies, 2010; Forslund et al., 2013). However, microbiota from isolated human populations have also been found to carry functional antibiotic resistance (AR) genes (Clemente et al., 2015). Soil has been implicated to be a key environmental reservoir of AR genes, as evidenced by the fact that AR genes with perfect nucleotide identity to those in human pathogens have been described in soil-dwelling bacteria (Forsberg et al., 2012).

In addition to soil, resistance genes have been found in a broad range of environments that contain complex microbial communities such as activated sludge (Mori et al., 2008; Munck et al., 2015), caves (Bhullar et al., 2012), glaciers (Segawa et al., 2013), rivers (Amos et al., 2014) and animals (Alves et al., 2014; Wichmann et al., 2014). As a result, all these environments, and probably many more, can act as potential reservoirs for resistance gene dissemination. It has been shown that AR is a natural phenomenon that predates the modern selective pressure of clinical antibiotic use (D'Costa et al., 2011), although little is known about the exact roles of these genes in their native environments (Sengupta et al., 2013). The marine environment is a major genetic reservoir of AR, which despite its tremendous bacterial diversity has been little studied in the context of AR gene dissemination (Yang et al., 2013). A recent study has identified a range of unclassified resistance genes in ocean water, thereby highlighting its importance as an environmental reservoir (Hatosy and Martiny, 2015).

Marine sponges are an ancient lineage of sessile filter-feeding metazoans. Many sponges harbor a dense and diverse microbiota comprising up to 40% of the total sponge volume, and as such, house a complex ecosystem characterized by host-microbe and microbe-microbe interactions (Webster and Taylor, 2012). Much sponge research has focused on the sponge microbiota as a source of novel bioactive compounds (Zhang et al., 2005), whereas its role as a reservoir of AR genes has received limited attention. Still, the fact that sponge microbiota can produce diverse antimicrobials (Laport et al., 2009; Mehbub et al., 2014) would suggest the presence of AR genes. An example of antimicrobials produced by sponge bacteria are rifampicin antibiotics, which at first were only known to be produced by soil *Actinobacteria* (August et al., 1998; Schupp et al., 1998), but more recently have also been found to be produced by a sponge-associated *Actinobacterium* sp. (Kim et al., 2006).

To date, research on functional AR genes in sponge microbiota has been limited to a *Bacillus* sp. isolated from the sponge *Haliclona simulans*. The *Bacillus* sp. was found to contain two small plasmids, one of which harbored the *tetL* tetracycline resistance gene (Phelan et al., 2011) whereas the other harbored the *ermT* erythromycin resistance gene (Barbosa et al., 2014). The plasmid with the *tetL* gene was shown to be nearly identical to three other tetracycline resistance plasmids identified in the honey bee pathogen *Paenibacilus larvae* pMA67, in the cheese-resident *Lactobacillus sakei* Rits 9, and in *Sporosarcina urea* pSU1 isolated from soil beneath a chicken farm (Phelan et al., 2011). Furthermore, a TEM β-lactamase-encoding gene was detected in a metatranscriptome dataset from the sponge *Crambe crambe* (Versluis et al., 2015), and the *mecA, mupA, qnrB*, and *tetL* resistance genes were detected in the sponge *Petromica citrina* by PCR with gene-specific primers (Laport et al., 2016). Based on these results it is tempting to speculate that the sponge microbiota indeed might act as a reservoir of functional AR genes.

The aim of this study was to systematically assess sponge microbiota as a reservoir of functional AR genes. We screened for functional resistance genes against 14 clinically relevant antibiotics in three Mediterranean high-microbial-abundance sponges, namely *Aplysina aerophoba, Petrosia ficiformis*, and *Corticium candelabrum*. Small-insert libraries were prepared in *Escherichia coli* with DNA isolated from sponge tissue of these three sponges (environmental DNA libraries), as well as from pooled DNA isolated from 31 bacterial isolates that were obtained from these sponges (sponge isolates DNA library) The 31 bacterial isolates were obtained from agar media supplemented with antibiotics as part of a previous high-throughput cultivation study (Versluis et al., under review).

## Materials and methods

### Resistance profiling of bacterial isolates

One small-insert library was prepared from genomic DNA obtained from 31 bacterial strains (Table 1) isolated from the sponges *A. aerophoba, C. candelabrum* and *P. ficiformis* (Supplementary Materials and Methods). Resistance of these isolates to polymyxin B, erythromycin, ciprofloxacin, cefotaxime, tetracycline, chloramphenicol, rifampicin, ampicillin and imipenem was investigated in a previous study (Supplementary Materials and Methods) (Versluis et al., under review). Additionally, in this study, resistance of the isolates was tested to gentamicin (50 μg/ml), D-cycloserine (50 μg/ml), chlortetracycline (50 μg/ml), amikacin (100 μg/ml) and trimethoprim (50 μg/ml).

**Table 1:** **Resistance profiles of bacterial strains isolated from the sponges *A. aerophoba, C. candelabrum* and *P. ficiformis***.

**Strain ID**	**Closest type strain (% identity)**	**Source**	**Accession**	**ChlTet**	**D-cycl**	**Gen**	**Ami**	**Trim**	**Pol**	**Ery**	**Cipro**	**Cefo**	**Tet**	**Chlo**	**Rif**	**Amp**	**Imi**
*Bacillus algicola* strain DN53_3H5	*B. algicola* (99.7)	*P. ficiformis*	KP769416					1									
*Bacillus idriensis* strain DN51_2A1	*B. idriensis* (99.9)	*A. aerophoba*	KP769417														
*Brachybacterium paraconglomeratum* strain DN73_5E10	*B. paraconglomeratum* (100)	*A. aerophoba*	KP769418														
*Brevibacterium* sp. DN213_3F7	*B. aurantiacum* (98.3)	*A. aerophoba*	KP769419														
*Microbulbifer* sp. DN217_4H2	*M. epialgicus* (98.9)	*C. candelabrum*	KP769420														
*Ruegeria atlantica* strain DN12_1A11	*R. atlantica* (99.2)	*A. aerophoba*	KP769421														
*Psychrobacter celer* strain DN193_4B9	*P. celer* (99.9)	*P. ficiformis*	KP769422					1									
*Janibacter melonis* strain DN216_4B10	*J. melonis* (99.3)	*A. aerophoba*	KP769423														
*Pseudovibrio ascidiaceicola* strain DN64_8G1	*P. ascidiaceicola* (99.6)	*P. ficiformis*	KP769424													1	
*Pseudovibrio ascidiaceicola* strain DN64_1D03	*P. ascidiaceicola* (99.6)	*A. aerophoba*	KP769425													1	
*Rhodococcus jialingiae* strain DN106_7C1	*R. jialingiae* (98.8)	*A. aerophoba*	KP769426														
*Acinetobacter radioresistens* strain DN138_5C8	*A. radioresistens* (100)	*A. aerophoba*	KP769427					1									
*Pseudovibrio* sp. DN49_8H4	*P. japonicus* (98.0)	*P. ficiformis*	KP769428					1									
*Non-labens arenilitoris* strain DN166_3E9	*N. arenilitoris* (99.6)	*A. aerophoba*	KP769429					1									
*Leisingera aquimarina* strain DN172_5F6	*L. aquimarina* (99.8)	*A. aerophoba*	KP769430														
*Flavobacteriaceae* sp. DN105_1H3	*M. aestuarii* (95.6)	*A. aerophoba*	KP769431					1									
*Ruegeria atlantica* strain DN83_2B6	*R. atlantica* (99.3)	*A. aerophoba*	KP769432														
*Pseudomonas oryzihabitans* strain DN90_5E11	*P. oryzihabitans* (99.7)	*C.candelabrum*	KP769433														
*Ruegeria* sp. DN110_6H4	*R. atlantica* (98.2)	*A. aerophoba*	KP769434														
*Bacillus* sp. DN88_4G3	*B. lentus* (98.0)	*P. ficiformis*	KP769435					1									
*Bacillus aryabhattai* strain DN67_5C7	*B. aryabhattai* (99.9)	*A. aerophoba*	KP769436												1	1	
*Aquimarina megaterium* strain DN30_1H2	*A. megaterium* (100)	*A. aerophoba*	KP769437					2									
*Sphingomonas* sp. DN81_6F7	*S. xenophaga* (100)	*P. ficiformis*	KP769438														
*Ruegeria* sp. DN71_7G3	*R. atlantica* (98.4)	*A. aerophoba*	KP769439														
*Bacillus horikoshii* strain DN9_1A9	*B. horikoshii* (99.9)	*A. aerophoba*	KP769440				1	1									
*Flavobacteriaceae* sp. DN50_6C1	*K. aquimaris* (94.9)	*A. aerophoba*	KP769441					1									
*Mycobacterium peregrinum* strain DN74_7A10	*M. peregrinum* (100)	*P. ficiformis*	KP769442														
*Bradyrhizobium pachyrhizi* strain DN55_6A7	*B. pachyrhizi* (99.3)	*A. aerophoba*	KP769443														
*Flavobacteriaceae* sp. DN112_6A5	*L. algicola* (96.7)	*A. aerophoba*	KP769444					1									
*Pseudovibrio* sp. DN206_4B7	*P. ascidiaceicola* (98.5)	*P. ficiformis*	KP769445														
*Bacillus stratosphericus* strain DN14_7A9	*B. stratosphericus* (99.9)	*P. ficiformis*	KP769446			2								1	1	1	

The strains were isolated and tested for antibiotic resistance in a previous study (Versluis et al, under review). We expanded the profile by also testing for resistance to chlortetracycline, D-cycloserine, gentamicin, amikacin and trimethoprim.

We defined three levels of antibiotic resistance: (i) “resistant”; growth of the bacteria was identical to their growth on media with no antibiotics, (ii) “intermediate resistance”; growth of the bacteria was slower than growth on media with no antibiotics, and (iii) “susceptible”; no growth. Dark green, light green and white indicate that the bacterium was respectively “resistant,” “intermediately resistant”, or “susceptible” to the antibiotic in question.

The accession numbers that link to 16S rRNA gene sequences of these strains are shown.

One small-insert library (library I-31) was prepared in E. coli with pooled genomic DNA from these bacteria. Numerical values indicate the number of times a resistance gene obtained from library I–31 (predicted with an E < 1.0E-7 i.e., at high confidence) was assigned to the strain providing resistance to the antibiotic in question.

### Preparation of linearized vector pZE21

An *E. coli* TOP10 strain harboring the plasmid pZE21 was inoculated into 10 ml LB broth with kanamycin (50 μg/ml). The culture was incubated at 37°C overnight. Plasmid isolation was performed with the GeneJET Plasmid Miniprep Kit (Thermo Fisher Scientific, Waltham, United States). The DNA concentration was measured by Qubit®; (Thermo Fisher Scientific), and the isolated plasmid was electrophoresed on a 1% agarose gel for visual inspection. Next, the plasmid was cut by HincII #R0103C (NEB, Ipswich, Massachusetts) according to manufacturer's instructions. Subsequently, the plasmid ends were dephosphorylated according to the protocol for dephosphorylation of 5′-ends of DNA by rSAP (NEB). Finally, the linearized and dephosphorylated plasmid DNA was purified with the Clean-up Concentrator kit (A&A Biotechnology, Gdynia, Poland).

### Preparation of small-insert libraries

To prepare a small-insert library with a mixture of genomic DNA from 31 pure culture isolates (Table 1), we individually isolated DNA with the MasterPure^™^ DNA Purification Kit (Epicentre, Madison, Wisconsin). The DNA concentration was measured by Qubit® 2.0, and DNA from the different isolates was then pooled at equivalent mass. DNA from sponge tissue samples was isolated with the DNeasy Blood & Tissue Kit (Qiagen, Hilden, Germany). DNA was fragmented by the E210 sonicator (Covaris, Woburn, Massachusetts) where default operating conditions were used to achieve an average fragment size of 2 kbp. The fragmented DNA was electrophoresed on a 1% agarose gel, and DNA ranging from 0.7 to 5 kbp was purified by using the GeneJet Gel Extraction Kit (Thermo Fisher Scientific). In the final gel purification step, DNA was eluted with nuclease-free water instead of elution buffer. Subsequently the DNA ends were repaired with the NEBNext® End Repair Module (NEB) according to manufacturer's instructions. The end-repaired DNA was cleaned with the Clean-up Concentrator kit (A&A Biotechnology), and elution was performed with nuclease-free water. The TaKaRa MightyMix DNA ligation kit (Clontech Laboratories Inc., Mountain View, United States) was used for ligation of the end-repaired fragments into the linearized pZE21 vector. Each ligation mixture consisted of 2 μl Ligation Mix and 2 μl nuclease-free water containing 600 ng insert DNA and 120 ng linearized vector (5:1 ratio). The resulting product was used for two electroporation reactions at 2 μl each, thereby creating two distinct small-insert libraries. At electroporation, 2 μl ligation mixture was added to 50 μl One Shot® TOP10 Electrocomp® *E. coli* cells (Thermo Fisher Scientific). The sample was transferred to a 1 mm cuvet and subjected to an electric pulse at 1.8 kV, 2.5 μF, and 200 Ω. To recover the *E. coli* cells, 1 ml SOC medium was added, and the sample was incubated at 37°C for 1 h. After recovery, 2 μl of the cell suspension was ten-fold serially diluted, and plated on LB agar media containing kanamycin (50 μg/ml) in order to estimate library size based on CFU counts. For each library, twelve clones were picked and colony PCRs were performed with primers pZE21_81_104_57C and pZE21_151_174rc_58C (Sommer et al., 2009), which flank the insertion site. The average product size (nt), the CFU count (in no. of colonies per μl) and the total volume (in μl) were multiplied in order to estimate the library size. In order to achieve library amplification, the remainder of the cell suspension after recovery was transferred to 10 ml LB medium with kanamycin (50 μg/ml) and incubated overnight at 37°C. After library amplification, another CFU count was performed. The total number of CFUs pre- and post-amplification was divided in order to estimate the extent of library expansion. Libraries were mixed to contain 20% glycerol and stored in cryotubes at −80°C. We also verified the function of a β-lactamase resistance gene by cloning it into the pZE21 vector as a single gene using the methods described in this paragraph, starting from the ligation step. The insert DNA consisted of amplicon sequences that were obtained by gene-specific PCR.

### Library screening and insert sequencing

Libraries were plated at 25X (predicted) library coverage (i.e., every clone is expected to be present 25 times) on LB agar plates containing one of the following antibiotics: ampicillin (20 μg/ml), ciprofloxacin (1 μg/ml), tetracycline (20 μg/ml), chloramphenicol (20 μg/ml), polymyxin B (2 μg/ml), trimethoprim (2 μg/ml), erythromycin (200 μg/ml), cefotaxime (25 μg/ml), rifampicin (25 μg/ml), imipenem (20 μg/ml), gentamicin (20 μg/ml), D-cycloserine (100 μg/ml), chlortetracycline (20 μg/ml) and amikacin (50 μg/ml). *E. coli* TOP10 cells without a plasmid were used as negative control. Inserts from antibiotic resistant clones were Sanger sequenced from both the 5′ and 3′ flanks with respectively the pZE21_81_104_57C and pZE21_151_174rc_58C primers. Clones were assumed to contain identical inserts if sequences were more than 99% identical over a stretch of >400 bp. One clone per set of identical clones was selected for further analysis. The full-length inserts of these representative clones were sequenced by primer walking. Sanger sequencing was performed by flanking primers custom-designed with Primer3Plus (Untergasser et al., 2007) until GeneStudio version 2.2.0.0 could assemble a contig that spanned the entire insert. Finally, vector sequences were removed by DNA Baser version 3.5.4.2.

### Analysis of inserts conferring resistance

ORFs were identified by MetaGeneMark (Zhu et al., 2010), and the corresponding nucleotide and derived amino acid sequences were extracted. Amino acid sequences were used for BLASTp (Altschul et al., 1990) against the NCBI non-redundant protein database and the CARD database (McArthur et al., 2013). BLASTn of the sequences against the NCBI nr/nt and CARD databases were performed as well. Resistance functions were also predicted with HMMER (http://hmmer.org/) using the profile hidden Markov models (pHMMs) of the Resfams database as a reference (Gibson et al., 2014). We defined a resistance gene to be identified with *high confidence* if it was detected at an E-value of <1E-7 by either BLASTp or BLASTn against the Comprehensive Antibiotic Resistance Database (CARD), or by employing the pHMMs of the Resfams database. In addition, the resistance function needed to match the observed resistance phenotype. InterProScan (Mitchell et al., 2015) was used for classifying proteins into families and for predicting the presence of domains. Clustal Omega (Sievers et al., 2011) was used to globally align resistance genes with their best hit in NCBI's non-redundant protein database and to calculate a global amino acid identity value. ISfinder (Siguier et al., 2006) was used to identify IS elements. The ACLAME server (Leplae et al., 2004) was used to investigate if parts of the insert sequences were previously identified on mobile genetic elements.

### Assignment of inserts conferring resistance to bacterial isolates

Since one small-insert library was prepared with genomic DNA from 31 different sponge-associated bacterial isolates (as opposed to metagenomic DNA), we could use insert sequence-specific PCRs to assign inserts to the bacterium of origin. For this purpose, primers were designed with Primer3Plus (Untergasser et al., 2007). The reaction mixture of a given detection PCR consisted of: 10 μl 5X Green GoTaq Reaction Buffer (Promega, Fitchburg, Wisconsin), 0.2 mM dNTPs (Promega), 1 μM forward primer, 1 μM reverse primer, 0.5 μl GoTaq G2 DNA polymerase (5 U/μl, Promega), 1 μl genomic DNA (10–20 ng/μl), and 32.5 μl nuclease-free water (Promega). The PCR program consisted of: initial denaturation of 30 s at 98°C; 35 cycles of denaturation at 98°C for 10 s, annealing at 57°C for 20 s, and extension at 72°C for 20 s; and final extension at 72°C for 10 min. The PCR product was analyzed on a 1% agarose gel. If neither the initial PCR nor a repetition with a 1°C reduction in annealing temperature yielded a product, then new primers were designed. If a detection PCR yielded a product with genomic DNA from multiple isolates, then the amplification products were sequenced by Sanger sequencing. Inserts were assigned to an isolate if the Sanger sequence was 99–100% identical to the insert sequence.

### Nucleotide sequence accession numbers

The metagenomic insert sequences were deposited under accession numbers KU577908 to KU577944. The insert that contained *bla*_PSV-1_ as a single gene was deposited under accession KU926347.

## Results

### Composition and screening of metagenomic libraries

In order to investigate the presence and diversity of functional AR genes in sponge microbiota, small-insert libraries were prepared in *E. coli* with a mixture of gDNA from 31 bacterial strains isolated from the sponges *A. aerophoba, P. ficiformis*, and *C. candelabrum* (library I-31; 0.8 Gb), and with DNA directly isolated from the same three sponges (libraries Aa; 0.2 Gb, Cc; 0.8 Gb, Pf; 1.7 Gb). 16S ribosomal RNA (rRNA) gene amplicon sequence analysis of the bacterial gDNA pool that was used to prepare library I-31 confirmed that DNA from all 31 isolates was present (data not shown). Amongst these 31 sponge-associated bacteria, resistance to 14 out of 16 antibiotics was observed, whereas merely intermediate resistance, signifying a reduced growth rate, to imipenem was observed. None of the isolates were resistant to rifampicin (Table 1). Screening of the four different libraries for AR yielded 37 clones with unique inserts conferring resistance to D-cycloserine (*n* = 6), gentamicin (*n* = 1), amikacin (*n* = 7), trimethoprim (*n* = 17), chloramphenicol (*n* = 1), rifampicin (*n* = 2) and ampicillin (*n* = 3) (Table 2). No clones were obtained that were resistant to chlortetracycline, polymyxin, erythromycin, ciprofloxacin, cefotaxime, tetracycline or imipenem. The majority of resistant clones (30 of 37) were derived from library I-31, whereas 2 clones, 0 clones and 5 clones were derived from libraries Aa, Cc, and Pf, respectively.

**Table 2:** **Small-insert libraries in *E. coli* were made with DNA isolated from 31 sponge bacteria, and with DNA isolated from *A. aerophoba, C. candelabrum* and *P. ficiformis* tissue**.

**Metagenomic library**	**Cumulative library size**	**D-cycloserine**	**Gentamicin**	**Amikacin**	**Trimethoprim**	**Chloramphenicol**	**Rifampicin**	**Ampicillin**
31 sponge bacteria (I-31)	0.8 Gb	6	1	4	13	1	2	3
*A. aerophoba* (Aa)	0.2 Gb	0	0	1	1	0	0	0
*C. candelabrum* (Cc)	0.8 Gb	0	0	0	0	0	0	0
*P. ficiformis* (Pf)	1.7 Gb	0	0	2	3	0	0	0

The libraries were screened for resistance to 14 antibiotics. This table shows the number of resistant clones with unique inserts for the different metagenomic libraries.

No clones were obtained that were resistant to chlortetracycline, polymyxin B, erythromycin, ciprofloxacine, cefotaxime, tetracycline, or imipenem.

### Resistance gene diversity and uniqueness

The unique full-length inserts (mean insert size = 2974 bp ± 1627 [s.d.]) were sequenced by Sanger sequencing, which occasionally required multiple iterations of primer walking. We defined a resistance gene to be identified with high confidence if the resistance function was assigned at an E-value of <1E-7, and furthermore the resistance function needed to match the observed resistance phenotype.

**Figure 1:**
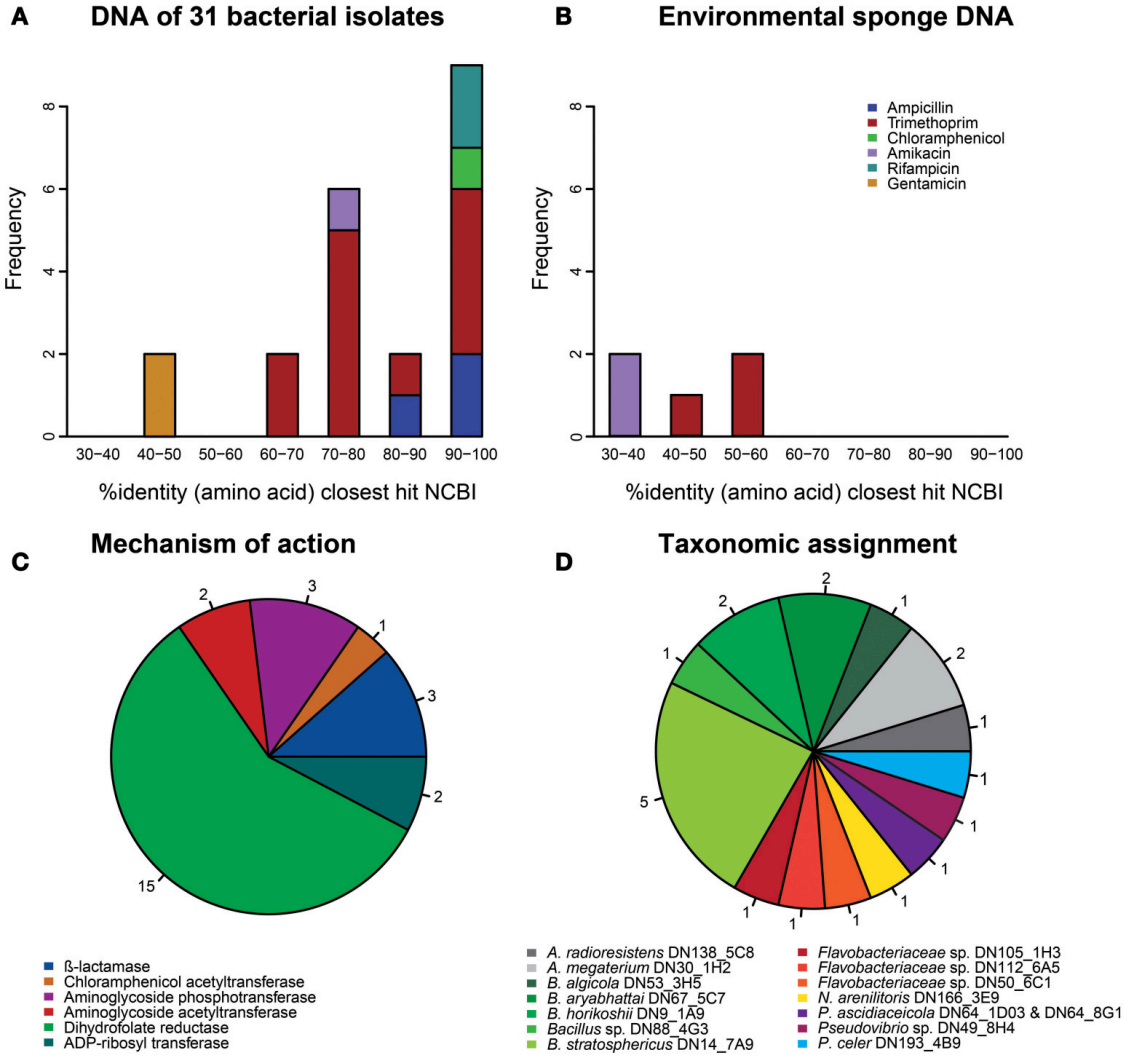
**Characterization of the 26 antibiotic resistance genes that were identified with high confidence (*E* < 1.0E-7). (A)** Amino acid identity distribution of the resistance genes that were obtained from the library of 31 sponge bacteria with their best hit (bitscore sorted) in NCBI's database. **(B)** Amino acid identity distribution of the resistance genes that were obtained from libraries based on DNA from sponge tissue with their best hit (bitscore sorted) in NCBI's database. **(C)** The mechanisms of action of all 26 resistance genes. **(D)** The taxonomic assignments of the resistance genes that were obtained from the library of 31 sponge bacteria.

In total, 26 resistance genes distributed over 24 different inserts were identified that met our confidence threshold (Table S1). The uniqueness of the resistance genes was evaluated by performing a global alignment at the amino acid level with the closest BLASTp hit in NCBI's non-redundant protein database. The majority of confidently identified resistance genes obtained from library I-31 (17 of 21) were predicted to code for proteins that had >70% identity at the amino acid level with known gene products, whereas proteins encoded by the genes (*n* = 5) obtained from the metagenomic libraries of environmental sponge DNA had <60% amino acid identity with known gene products (Figures 1A,B). Overall, most confidently identified resistance genes (15/26) were predicted to encode trimethoprim-resistance-conferring dihydrofolate reductases (Figure 1C). The confidently identified AR genes with the lowest similarity to known genes were predicted to code for a glycerol-3-phosphate acyltransferase and a GNAT family acetyltransferase with respectively 32 and 36% amino acid identity with the closest hit in NCBI's non-redundant protein database. Both of these amikacin-resistance-conferring genes were detected in libraries of environmental sponge DNA.

For 13 insert sequences a resistance gene could not be identified meeting our threshold. Those inserts conferred resistance to amikacin (*n* = 4), D-cycloserine (*n* = 6), and trimethoprim (*n* = 3) (Table 3, Table S2). In these cases, besides BLAST searches against the CARD and NCBI nr/nt databases and motif detection by application of Resfams pHMMs, also protein domain and protein family predications by InterProScan were evaluated in order to manually predict AR gene presence. Most (12 of 13) inserts contained multiple open reading frames (ORFs), which prevented the unequivocal assignment of resistance functions to individual genes. For three of four inserts that conferred resistance to amikacin, we predicted the presence of genes encoding aminoglycoside-modifying enzymes. On one of these inserts (clone Env_Ami3) a gene was identified that we predicted to code for an enzyme that putatively modifies amikacin by phosphorylation. On the other two inserts, we predicted genes encoding an aminoglycoside acetyltransferase (clone Iso_Ami2) and an aminoglycoside methyltransferase (clone Iso_Ami3). The fourth insert conferring amikacin resistance (clone Iso_Ami4) contained a single gene that shared 93% amino acid identity with an amino acid transporter, and as such we predicted the gene to confer AR via antibiotic efflux. Six inserts conferred resistance to D-cycloserine. On all of these six inserts we predicted the presence of genes encoding proteins involved in transmembrane transport based on high similarity to known transporters. We predicted that three of the six D-cycloserine resistance-conferring inserts harbored genes encoding transporters that belong to the major facilitator superfamily. Three other inserts on which an AR gene could not be confidently identified conferred resistance to trimethoprim. On one of these inserts (clone Env_Trim4), we predicted that a thymidylate synthethase may be responsible for the resistance phenotype. The two other inserts conferring trimethoprim resistance (clones Iso_trim13 and Iso_trim14) putatively encoded oxidoreductases that could confer trimethoprim resistance.

**Table 3:** **Inserts that confer resistance to amikacin, D-cycloserine or trimethoprim on which no AR gene was identified with high confidence**.

**Clone ID**	**Clone accession**	**Library**	**Resistance**	**Gene**	**Annotation best hit in NCBI nr/nt**	**% amino acid identity**	**Predicted resistance function**
Iso_Ami2	KU577920	I-31	Amikacin	2..934	Flagellar motor protein [*Ruegeria* sp. ANG-R]	92.3	Aminoglycoside 6′-N-acetyltransferase
				1,043..1,687	Hypothetical protein [*Ruegeria* sp. ANG-S4]	57.9	
				1,808..2,197	ATP-dependent Clp protease ATP-binding subunit ClpA [*Ruegeria* sp. CECT 5091]	98.5	
				2,293..3,741	MULTISPECIES: copper amine oxidase [*Bacillus*]	99.6	
Iso_Ami3	KU577921	I-31	Amikacin	2..526	16S rRNA (guanine(966)-N(2))-methyltransferase RsmD [*Pseudovibrio* sp. POLY-S9]	98.9	Aminoglycoside methyltransferase
				1,661..2,977	Transporter [*Psychrobacter aquaticus*]	86.1	
Iso_Ami4	KU577922	I-31	Amikacin	1..1,329	Amino acid transporter [*Pseudovibrio* sp. FO-BEG1]	92.7	Transmembrane export
Env_Ami3	KU577939	Pf	Amikacin	2..73	n/a	n/a	
				388..1,005	Non-canonical purine NTP pyrophosphatase [*Thermogemmatispora carboxidivorans*]	49.1	Aminoglycoside modification
				1,086..1,382	Radical SAM protein [*Deinococcus radiodurans*]	59.6	
Iso_Dcy1	KU577913	I-31	D-cycloserine	1..543	GntR family transcriptional regulator [*Bacillus altitudinis*]	99.5	
				593..910	Major facilitator superfamily transporter [*Bacillus stratosphericus* LAMA 585]	94.3	Antibiotic efflux
				903..1,760	MFS transporter [*Bacillus* sp. TH007]	95.8	Antibiotic efflux
				1,928..2,206	Barnase inhibitor [*Bacillus aerophilus*]	15.9	
				2,363..2,785	MULTISPECIES: iron ABC transporter permease [*Bacillus*]	100	
Iso_Dcy2	KU577914	I-31	D-cycloserine	2..199	N-acetylmuramic acid 6-phosphate etherase [*Bacillus manliponensis*]	72.7	
				333..515	Hypothetical protein [*Bacillus fordii*]	73.7	
				618..1,838	Hypothetical protein [*Bacillus fordii*]	55.6	Antibiotic efflux
				1,955..2,323	NADPH:quinone oxidoreductase [*Aneurinibacillus tyrosinisolvens*]	74.0	
Iso_Dcy3	KU577915	I-31	D-cycloserine	3..1,214	GntR family transcriptional regulator [*Bacillus* sp. FJAT-21351]	100	
				1,365..1,586	EamA-like transporter family, partial [uncultured bacterium]	82.2	Antibiotic efflux
				1,637..2,284	MULTISPECIES: multidrug transporter [*Bacillus*]	100	
Iso_Dcy4	KU577916	I-31	D-cycloserine	437..847	Transporter [*Bacillus megaterium*]	93.4	Antibiotic efflux
				916..1,596	Transporter [*Bacillus megaterium*]	99.5	Antibiotic efflux
				2,031..3,158	MFS transporter [*Bacillus megaterium*]	99.7	Antibiotic efflux
Iso_Dcy5	KU577917	I-31	D-cycloserine	15..893	Transporter [*Pseudovibrio* sp. POLY-S9]	99.3	Antibiotic efflux
				944.1,216	Succinate dehydrogenase [*Pseudovibrio* sp. POLY-S9]	98.9	
				1,203..1,946	Succinate dehydrogenase [*Pseudovibrio* sp. POLY-S9]	91.9	
Iso_Dcy6	KU577918	I-31	D-cycloserine	1..90	MULTISPECIES: hypothetical protein [*Rhodococcus*]	100	
				95..259	MULTISPECIES: hypothetical protein [*Rhodococcus*]	69.9	
				409..924	RTX toxin [*Rhodobacteraceae* bacterium KLH11]	70.9	
				926..1,576	RTX toxin [*Ruegeria conchae*]	78.7	
				1,752..2,105	Hypothetical protein RHECNPAF_930033 [*Rhizobium etli* CNPAF512]	28.2	
				2,102..2,362	Membrane protein [*Acinetobacter baumannii*]	100	
				2,467..3,309	Putative benzoate transporter [*Acinetobacter radioresistens* DSM 6976]	99.6	Antibiotic efflux
Iso_Trim13	KU577923	I-31	Trimethoprim	3..176	Sodium:proton exchanger [*Ruegeria* sp. 6PALISEP08]	87.7	
				246..977	Short-chain dehydrogenase [*Ruegeria halocynthiae*]	84.9	Oxidoreductase
				1,268..3,073	Excinuclease ABC subunit C [*Ruegeria* sp. CECT 5091]	93.3	
				3,143..3,685	Membrane protein [*Ruegeria halocynthiae*]	74.4	
				3,986..4,492	Hypothetical protein [*Microbulbifer variabilis*]	91.1	
Iso_Trim14	KU577937	I-31	Trimethoprim	3..440	Nitrous-oxide reductase [*Lacinutrix himadriensis*]	83.5	Oxidoreductase
				379..828	Nitrous-oxide reductase [*Gaetbulibacter saemankumensis*]	85.1	Oxidoreductase
				800..1,279	Nitrous-oxide reductase [*Gaetbulibacter saemankumensis*]	83.2	Oxidoreductase
				1,858..2,130	Membrane protein [*Flaviramulus ichthyoenteri*]	80.2	
				2,096..2,410	Hypothetical protein [*Mesoflavibacter zeaxanthinifaciens*]	77.8	
Env_Trim4	KU577943	Aa	Trimethoprim	3..521	Td thymidylate synthetase *[Synechococcus* phage Syn19]	80.8	Thymidylate synthetase
				586..882	Hypothetical protein SSSM7_321 [*Synechococcus* phage S-SSM7]	75.3	
				884..1,273	P-starvation inducible protein [uncultured Mediterranean phage uvMED]	81.5	

We defined a resistance gene to be identified with high confidence if it was detected at an E-value of <1E-7 by either BLASTp or BLASTn against the CARD database, or by employing the pHMMs of the Resfams database. However, on these inserts no resistance genes were identified with high confidence. Therefore, in order to predict the presence of resistance genes, the results from the queries against the CARD and Resfams database were supplemented with protein domain and protein family predications by InterProScan. Based on the combined results, we predicted which genes on these inserts may be responsible for the resistance phenotype. The column “predicted resistance function” contains information about the mechanism of action of the proteins that we predicted to be responsible for the resistance phenotype.

### Taxonomic assignment of inserts from the library of 31 sponge bacteria

Resistance genes that were confidently identified in library I-31 were assigned to their strain of origin by gene-specific PCRs, where the PCR amplicon covered at least part of the predicted resistance gene. Gene presence was checked in all 31 strains used for building the library by dedicated PCR reactions, which resulted in confidently identified AR genes being assigned to 15 of 31 bacterial strains in library I-31 (Table 1). Insert sequences for which no resistance gene could be identified with high confidence were assigned to a strain of origin at the loci given in Table S2. We discovered that several inserts may contain hybrid sequences, that is, the inserts consist of two different DNA fragments that were ligated into the vector. For example, the insert of clone Iso_Dcy6 was assigned to *Ruegeria* spp. in the region between 513 and 1372 bp and assigned to *Acinetobacter radioresistens* DN138_5C8 in the region between 2240 and 2851 (Table S2). We found that about half of the confidently identified resistance genes (11/21) were assigned to *Bacillus* spp., and *Bacillus* was the only taxon to which gentamicin, amikacin, chloramphenicol and rifampicin resistance genes were assigned (Table 1, Figure 1D). Even though gentamicin, amikacin, chloramphenicol, rifampicin and ampicillin resistance genes were assigned to *Bacillus* spp., the strains themselves were not found to be resistant to these antibiotics. Resistance genes that were assigned to the remaining taxa comprised mostly trimethoprim resistance genes, with an exception being an ampicillin resistance gene that was assigned to *Pseudovibrio* spp. (Table S1). Strains to which trimethoprim resistance genes were assigned were not trimethoprim resistant themselves in 8 out of 9 cases.

### Novel β-lactamase family identified in *Pseudovibrio*

**Figure 2:**
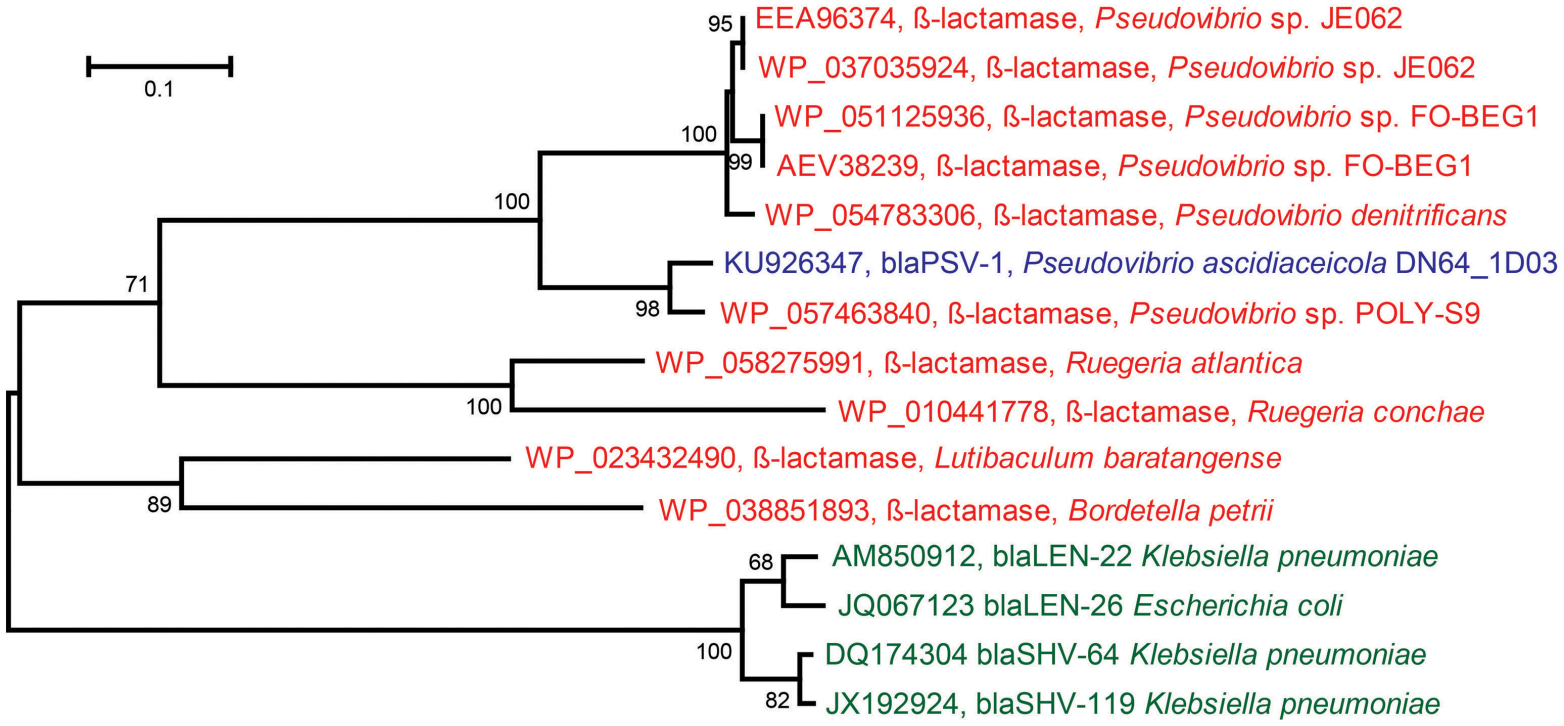
**Maximum Likelihood Tree based on protein sequences of: the novel ß-lactamase (*bla*_PSV-1_) discovered in *Pseudovibrio ascidiaceicola* DN64_1D03 (blue), the closest homologs of the novel ß-lactamase in the NCBI non-redundant protein sequences database (red), and the closest homologs of the novel ß-lactamase in the CBMAR database (green) (the two closest homologs were picked for each of the two closest families: *bla*_LEN_ and *bla*_SHV_)**. In this figure, only the proteins in green and the novel ß-lactamase (*bla*_PSV-1_) have demonstrated functionality. The tree was constructed in MEGA using 1000 iterations of bootstrapping. Bootstrap values <50 are not shown. The horizontal bar indicates the number of substitutions per site.

The insert sequence of ampicillin-resistant clone Iso_Amp3 contained a gene predicted to encode a class A β-lactamase. The gene was assigned to *Pseudovibrio ascidiaceicola* DN64_1D03 and *Pseudovibrio ascidiaceicola* DN64_8G1, and the predicted protein shared 58% amino acid identity with the closest hit in the CBMAR database (Srivastava et al., 2014). Since this predicted protein displayed high divergence with members of known β-lactamase families it is a candidate to be classified into a new family (Jacoby, 2006). Therefore, we further tested the gene's function by cloning the gene-specific amplicon sequence that was generated with genomic DNA of *Pseudovibrio ascidiaceicola* DN64_1D03 as template into the pZE21 vector. The new clone, PSV1 (accession KU926347), was resistant to ampicillin, which confirmed the functionality of the β-lactamase gene. Remarkably, the coding sequence of the gene cloned using the genome-derived amplicon sequences was 75 bp longer than the coding sequence of the β-lactamase gene in the original metagenomic insert. Sanger sequencing of the genome-derived amplicon yielded a gene sequence that shared 100% nucleotide identity with the gene sequence in clone PSV1. Hence, we concluded that the longer β-lactamase gene in clone PSV1 is the exact gene that is present in the genome of *Pseudovibrio ascidiaceicola* DN64_1D03. We predict that even though a frameshift mutation yielded a shorter gene in case of the small-insert library, the encoded enzyme retained its function. We classified the genome-derived (longer) gene into a novel β-lactamase family named PSV after *Pseudovibrio*. The first member was designated *bla*_PSV-1_. BLASTp of the gene against the CBMAR database showed that LEN and SHV were the closest β-lactamase families. The best hit from the LEN and SHV families in both cases shared 50% amino acid identity with *bla*_PSV-1_. The best hit was *bla*_LEN−22_ (accession AM850912), with which *bla*_PSV-1_shared 41% global amino acid identity. The best hits in the NCBI non-redundant protein sequences database (≥76% amino acid identity) were exclusively genes in sequenced genomes of *Pseudovibrio* spp. (Figure 2). Additional resistance testing of clone PSV1 showed that *bla*_PSV-1_ conferred resistance to penicillin (50 μg/ml) but not against cefotaxime (20 μg/ml) and imipenem (20 μg/ml).

### Resistance gene dissemination

Horizontal gene transfer is the main mechanism through which resistance genes are disseminated. Therefore, we investigated our sequences for evidence of gene mobilization. Comparison of the insert sequences with the ACLAME plasmid database showed that they were not previously identified on plasmids. Furthermore, ISfinder did not identify insertion sequences. We investigated whether the resistance genes identified in this study had been observed previously in a different genomic context by comparing our sequences with those in the NCBI nr/nt database (Table S3). A β-lactamase and an ADP-ribosyl transferase (those on clones Iso_Amp2 and Iso_Rif2, respectively), both of which were assigned to *Bacillus aryabhattai* DN67_5C7, shared very high (≥99%) identity with genes previously found in the non-marine *Bacillus megaterium* QM B1551, which suggests a relatively recent dissemination event. The insert of clone Iso_Amp2 shared 99% identity with the genome of *Bacillus megaterium* QM B1551 over the full-length insert (1352 bp). This insert produced four other significant alignments with sequences in the NCBI nr/nt database at >90% nucleotide identity, all of which belonged to *Bacillus megaterium* strains. The insert of clone Iso_Rif2 was 6717 bp in length but only the region from 5198 to 6717 bp aligned with the genome of *Bacillus megaterium* QM B1551. Besides the rifampicin-resistance-conferring gene, this region encodes a hypothetical protein and a gene that putatively encodes an enzyme involved in site specific recombination of plasmids. The gene putatively encoding a recombination enzyme was the only gene involved in gene mobilization that was predicted on the inserts.

## Discussion

Functional metagenomic analysis of sponge-associated bacteria revealed diverse AR genes that conferred resistance to ampicillin, D-cycloserine, gentamicin, amikacin, trimethoprim, chloramphenicol and rifampicin. Seventeen of 26 confidently identified AR genes shared low homology (<90% amino acid identity) with known gene products. Therefore, these results show that sponges, like other environments that contain complex bacterial communities, appear to represent reservoirs of unique AR genes. In addition, we obtained 13 inserts that did not contain genes with significant similarity to known resistance genes, reinforcing the notion that sponges may act as reservoirs of yet unknown mechanisms of AR. Two resistance genes shared ≥99% nucleotide identity with AR genes detected outside the marine environment, and hence it is tempting to speculate that AR genes are being transmitted between sponges and non-marine environments and as such may be exploited by bacteria in other non-marine habitats. It has previously been shown that AR genes can be genetically linked, leading to co-selection (Galimand et al., 1997; Summers, 2006). None of our inserts contained AR genes with different resistance functions, which is likely in part due to the small insert size of the metagenomic libraries.

Out of 37 inserts harboring resistance genes, 30 inserts were obtained from the library of 31 sponge bacteria, and 7 inserts were obtained from the libraries of sponge tissue even though their cumulative library sizes were 0.8 and 2.7 Gbp, respectively. We expect that this discrepancy can be explained by libraries from sponge tissue DNA containing a substantial fraction of sponge DNA. No identical resistance genes were obtained from individual isolates and from sponge tissue in spite of the individual isolates being previously isolated from the tissue samples. This result, and taking into account the high AR gene diversity and limited scale of the experiment, suggests the presence of a substantial number of AR genes in sponges that are not yet identified. The number of AR genes that we identified was also restricted to those that are compatible with the expression system of the *E. coli* host, and to those that confer AR based on a DNA sequence of at most 5 kbp. From the sponge *C. candelabrum* no resistant clones were obtained, neither from the sponge tissue nor from the individual isolates. It should be noted, however, that the small-insert library of 31 bacteria contained only two isolates from *C. candelabrum*, which reduced the chance to identify a resistance gene from this sponge. Furthermore, the library of *C. candelabrum* tissue was 0.8 Gb in size and as such is expected to cover only a small fraction of the total unique DNA in this niche. Therefore, absence of resistance inserts for this sponge species should not be interpreted as an absence of AR genes.

Most confidently identified resistance genes (15/26) conferred resistance to trimethoprim by encoding the enzyme dihydrofolate reductase. Dihydrofolate reductase is a housekeeping enzyme that converts dihydrofolic acid to tetrahydrofolic acid. Tetrahydrofolic acid in turn is a required cofactor to convert dUMP to dTMP by thymidylate synthase, with dTMP being essential for DNA synthesis. Trimethoprim resistance due to additional dihydrofolate reductase genes can occur via a combination of two mechanisms: (i) the dihydrofolate reductase is overproduced leading to higher levels of the enzyme in the cell, and (ii) the heterologously introduced dihydrofolate reductase has decreased trimethoprim susceptibility due to mutations (Gibreel and Sköld, 1998; Coque et al., 1999; Rodrigues et al., 2016). The fact that strains to which dihydrofolate reductase genes were assigned were themselves in most cases (8/9) not trimethoprim resistant may have resulted from these strains having too little active dihydrofolate reductase to compensate for the inhibition by trimethoprim. Resistance in recombinant *E. coli* clones that host a plasmid-encoded dihydrofolate reductase may result from 50 to 70 copies of pZE21 plasmid being present per cell (Lutz and Bujard, 1997), thereby substantially increasing the concentration of active enzyme. Expression level differences of active enzyme could also explain other instances where the host to which an AR gene was assigned was itself not resistant. For example, *Bacillus stratosphericus* DN14_7A9 contained functional genes conferring resistance to gentamicin, chloramphenicol, rifampicin and ampicillin, but the strain itself was not resistant against these antibiotics. In the natural environment, these AR genes can potentially benefit their bacterial hosts in the presence of lower levels of antimicrobials than those applied in this study, or even if antimicrobials are present at sub-MIC levels (Andersson and Hughes, 2012; Sandegren, 2014). For example, an *Escherichia coli* strain harboring a single *GyrA* mutation was shown to outcompete the wild-type strain at a ciprofloxacin level of 1/230 the wild-type MIC (Gullberg et al., 2011).

The most unique confidently identified (predicted) resistance genes were those encoding a glycerol-3-phosphate acyltransferase and a GNAT family acetyltransferase with respectively 32 and 36% amino acid identity with the closest hit in NCBI's non-redundant protein database. These genes were derived from libraries Aa and Pf, respectively. Although aminoglycoside resistance genes might serve as defense against locally or self-produced antimicrobials (Walsh and Duffy, 2013), past studies have ascribed to these genes various other functional roles besides aminoglycoside modification. Aminoglycoside modification would then be accidental. For instance, a 2′-N-acetyltransferase was shown to be involved in the acetylation of peptidoglycan (Macinga et al., 1998). Reeves et al. (2013) suggested roles for aminoglycoside resistance genes in immune modulation and alleviation of cellular stress. In addition, aminoglycoside-3′-phosphotransferases are closely related to protein kinases (Davies, 2006). For one amikacin-resistance conferring insert, we predicted that resistance resulted from antibiotic efflux. The efflux pump showed high identity with an amino acid transporter that was not previously implicated in amikacin resistance. For another six metagenomic inserts from our libraries that conferred D-cycloserine resistance, we predicted the presence of efflux pumps that were also not previously linked to resistance. These results show that sponge-derived bacteria carry genes that provide AR by mechanisms that were not previously observed.

One novel β-lactamase (*bla*_PSV-1_) was discovered in *P. ascidiaceicola* DN64_1D03 that provided resistance to ampicillin and penicillin. Based on high amino acid sequence divergence with known β-lactamase families, the gene was placed in a novel family termed PSV, after *Pseudovibrio*. To date, all publicly available sequences that share ≥76% identity with *bla*_PSV-1_ at the amino acid level were found in *Pseudovibrio* spp. (Figure 2), a genus whose members are consistently being isolated from sponges and have never been isolated outside the marine environment (Lafi et al., 2005; Muscholl-Silberhorn et al., 2008; Menezes et al., 2010). The subsequent closest homologs of the novel β-lactamase were (predicted) β-lactamases from *Ruegeria*, a genus comprising marine bacteria (Wagner-Döbler and Biebl, 2006). Therefore, *bla*_*PSV*-1_ appears to be a marine-specific β-lactamase, and may correspondingly have a marine-specific role and/or substrate. In sponges, β-lactamases could serve as a defense molecule against β-lactam antimicrobials, and substances containing a β-lactam ring have been identified in sponges (Aviles and Rodriguez, 2010). On the other hand, β-lactamases have also been predicted to serve a role in disruption of cell signaling (Allen et al., 2009). Apart from the biological role of β-lactamases, their evolutionary origin is also still unclear (Hall and Barlow, 2004; Garau et al., 2005).

The majority of confidently identified AR genes (11/21) in library I-31 were assigned to *Bacillus* spp. including AR genes conferring resistance to ampicillin, chloramphenicol, gentamicin, amikacin and rifampicin. This is in line with the fact that, to date, the only sponge isolate that was shown to harbor functional resistance genes was *Bacillus* sp. strain HS24 (Phelan et al., 2011; Barbosa et al., 2014). These findings suggest that in sponges, *Bacillus* spp. are a reservoir of AR genes. However, sponges filter thousands of liters of water per day (Vogel, 1977), and as a result, sponge-associated microorganisms are not always permanent residents of the sponge holobiont. In fact, in all three investigated sponges *Bacillus* spp. represent less than 0.1% of the sponge microbiome (Versluis et al., under review). Therefore, we speculate that the diverse AR genes detected in the sponge-associated *Bacillus* spp. do not play a sponge-specific role but rather that these genes serve a more general role in the survival of these bacteria in the marine environment.

A phenomenon we noticed is that in our study, several inserts may contain hybrid sequences, that is, the inserts consist of two different DNA fragments that were ligated into the same vector. As a consequence, the assignment of inserts to a strain of origin can depend on the sequence locus that is amplified by detection PCR. We suspect that these hybrid inserts are an artifact of the cloning strategy involving ligation of blunt end fragments. Because in the present study, one of the libraries was prepared from DNA extracted from 31 bacterial isolates, and thus a defined pool of starting DNA was used, detection of hybrid inserts was possible. However, based on this finding, for all studies using similar cloning methods conclusions about the genetic context of AR genes on metagenomic inserts should be drawn carefully. Here, to ensure that all confidently identified resistance genes were assigned to the correct strain, we performed gene-specific PCRs (Table S1).

In conclusion, we show that sponges constitute a reservoir of diverse functional AR genes. We detected functional AR genes that show little similarity to known AR genes, as well as functional AR genes that have no similarity to known AR genes. One ampicillin resistance gene, *bla*_PSV-1_, was placed into a novel family of marine-specific β-lactamases. These results raise questions as to the roles of these genes in bacteria residing in arguably the oldest lineage of metazoans (Hentschel et al., 2006), the sponges. Furthermore, the functionality of our observed AR genes in *E. coli* shows that they can potentially be harnessed by phylogenetically distinct bacteria in other environments.

## Author contributions

DV was involved in experimental design, performed the experiments and the analysis, wrote the manuscript, and prepared the figures. MV, HS, and DS were involved in experimental design and jointly supervised the work. MR carried out part of the experimental work. All authors revised the manuscript.

### Conflict of interest statement

The authors declare that the research was conducted in the absence of any commercial or financial relationships that could be construed as a potential conflict of interest.

## References

[B1] Allen H. K. Moe L. A. Rodbumrer J. Gaarder A. Handelsman J. (2009). Functional metagenomics reveals diverse β-lactamases in a remote Alaskan soil. ISME J. 3, 243–251. 10.1038/ismej.2008.86 18843302

[B2] Altschul S. F. Gish W. Miller W. Myers E. W. Lipman D. J. (1990). Basic local alignment search tool. J. Mol. Biol. 215, 403–410. 10.1016/S0022-2836(05)80360-2 2231712

[B3] Alves M. S. Pereira A. Araújo S. M. Castro B. B. Correia A. C. Henriques I. (2014). Seawater is a reservoir of multi-resistant *Escherichia coli*, including strains hosting plasmid-mediated quinolones resistance and extended-spectrum beta-lactamases genes. Front. Microbiol. 5:426. 10.3389/fmicb.2014.00426 25191308 PMC4138442

[B4] Amos G. C. Zhang L. Hawkey P. M. Gaze W. H. Wellington E. M. (2014). Functional metagenomic analysis reveals rivers are a reservoir for diverse antibiotic resistance genes. Vet. Microbiol. 171, 441–447. 10.1016/j.vetmic.2014.02.017 24636906

[B5] Andersson D. I. Hughes D. (2012). Evolution of antibiotic resistance at non-lethal drug concentrations. Drug Resist. Updat. 15, 162–172. 10.1016/j.drup.2012.03.005 22516308

[B6] Arias C. A. Murray B. E. (2009). Antibiotic-resistant bugs in the 21st century–a clinical super-challenge. N. Engl. J. Med. 360, 439–443. 10.1056/NEJMp0804651 19179312

[B7] August P. R. Tang L. Yoon Y. J. Ning S. Müller R. Yu T. W.. (1998). Biosynthesis of the ansamycin antibiotic rifamycin: deductions from the molecular analysis of the rif biosynthetic gene cluster of *Amycolatopsis mediterranei* S699. Chem. Biol. 5, 69–79. 10.1016/S1074-5521(98)90141-7 9512878

[B8] Aviles E. Rodriguez A. D. (2010). Monamphilectine A, a potent antimalarial β-lactam from marine sponge *Hymeniacidon* sp: isolation, structure, semisynthesis, and bioactivity. Org. Lett. 12, 5290–5293. 10.1021/ol102351z 20964325 PMC2982677

[B9] Barbosa T. M. Phelan R. W. Leong D. Morrissey J. P. Adams C. Dobson A. D.. (2014). A novel erythromycin resistance plasmid from *Bacillus* sp. strain HS24, isolated from the marine sponge *Haliclona simulans*. PLoS ONE 9:e115583. 10.1371/journal.pone.0115583 25548909 PMC4280177

[B10] Bhullar K. Waglechner N. Pawlowski A. Koteva K. Banks E. D. Johnston M. D.. (2012). Antibiotic resistance is prevalent in an isolated cave microbiome. PLoS ONE 7:e34953. 10.1371/journal.pone.0034953 22509370 PMC3324550

[B11] Clemente J. C. Pehrsson E. C. Blaser M. J. Sandhu K. Gao Z. Wang B.. (2015). The microbiome of uncontacted Amerindians. Sci. Adv. 1:e1500183. 10.1126/sciadv.1500183 26229982 PMC4517851

[B12] Coque T. M. Singh K. V. Weinstock G. M. Murray B. E. (1999). Characterization of dihydrofolate reductase genes from trimethoprim-susceptible and trimethoprim-resistant strains of *Enterococcus faecalis*. Antimicrob. Agents Chemother. 43, 141–147. 9869579 10.1128/aac.43.1.141 PMC89034

[B13] Davies J. Davies D. (2010). Origins and evolution of antibiotic resistance. Microbiol. Mol. Biol. Rev. 74, 417–433. 10.1128/MMBR.00016-10 20805405 PMC2937522

[B14] Davies J. E. (2006). Aminoglycosides: ancient and modern. J. Antibiot. 59, 529–532. 10.1038/ja.2006.73 17136885

[B15] D'Costa V. M. King C. E. Kalan L. Morar M. Sung W. W. Schwarz C.. (2011). Antibiotic resistance is ancient. Nature 477, 457–461. 10.1038/nature10388 21881561

[B16] Forsberg K. J. Reyes A. Wang B. Selleck E. M. Sommer M. O. Dantas G. (2012). The shared antibiotic resistome of soil bacteria and human pathogens. Science 337, 1107–1111. 10.1126/science.1220761 22936781 PMC4070369

[B17] Forslund K. Sunagawa S. Kultima J. R. Mende D. R. Arumugam M. Typas A.. (2013). Country-specific antibiotic use practices impact the human gut resistome. Genome Res. 23, 1163–1169. 10.1101/gr.155465.113 23568836 PMC3698509

[B18] Galimand M. Guiyoule A. Gerbaud G. Rasoamanana B. Chanteau S. Carniel E.. (1997). Multidrug resistance in *Yersinia pestis* mediated by a transferable plasmid. N. Engl. J. Med. 337, 677–680. 10.1056/NEJM199709043371004 9278464

[B19] Garau G. Di Guilmi A. M. Hall B. G. (2005). Structure-based phylogeny of the metallo-β-lactamases. Antimicrob. Agents Chemother. 49, 2778–2784. 10.1128/AAC.49.7.2778-2784.2005 15980349 PMC1168685

[B20] Gibreel A. Sköld O. (1998). High-level resistance to trimethoprim in clinical isolates of *Campylobacter jejuni* by acquisition of foreign genes (dfr1 and dfr9) expressing drug-insensitive dihydrofolate reductases. Antimicrob. Agents Chemother. 42, 3059–3064. 9835491 10.1128/aac.42.12.3059 PMC105999

[B21] Gibson M. K. Forsberg K. J. Dantas G. (2014). Improved annotation of antibiotic resistance determinants reveals microbial resistomes cluster by ecology. ISME J. 9, 207–216. 10.1038/ismej.2014.106 25003965 PMC4274418

[B22] Gullberg E. Cao S. Berg O. G. Ilbäck C. Sandegren L. Hughes D.. (2011). Selection of resistant bacteria at very low antibiotic concentrations. PLoS Pathog. 7:e1002158. 10.1371/journal.ppat.1002158 21811410 PMC3141051

[B23] Hall B. G. Barlow M. (2004). Evolution of the serine β-lactamases: past, present and future. Drug Resist. Updat. 7, 111–123. 10.1016/j.drup.2004.02.003 15158767

[B24] Hatosy S. M. Martiny A. C. (2015). The Ocean as a global reservoir of antibiotic resistance genes. Appl. Environ. Microbiol. 81, 7593–7599. 10.1128/AEM.00736-15 26296734 PMC4592852

[B25] Hentschel U. Usher K. M. Taylor M. W. (2006). Marine sponges as microbial fermenters. FEMS Microbiol. Ecol. 55, 167–177. 10.1111/j.1574-6941.2005.00046.x 16420625

[B26] Högberg L. D. Heddini A. Cars O. (2010). The global need for effective antibiotics: challenges and recent advances. Trends Pharmacol. Sci. 31, 509–515. 10.1016/j.tips.2010.08.002 20843562

[B27] Jacoby G. A. (2006). β-lactamase nomenclature. Antimicrob. Agents Chemother. 50, 1123–1129. 10.1128/AAC.50.4.1123-1129.2006 16569819 PMC1426973

[B28] Kim T. K. Hewavitharana A. K. Shaw P. N. Fuerst J. A. (2006). Discovery of a new source of rifamycin antibiotics in marine sponge *actinobacteria* by phylogenetic prediction. Appl. Environ. Microbiol. 72, 2118–2125. 10.1128/AEM.72.3.2118-2125.2006 16517661 PMC1393243

[B29] Lafi F. F. Garson M. J. Fuerst J. A. (2005). Culturable bacterial symbionts isolated from two distinct sponge species (*Pseudoceratina clavata* and *Rhabdastrella globostellata*) from the Great Barrier Reef display similar phylogenetic diversity. Microb. Ecol. 50, 213–220. 10.1007/s00248-004-0202-8 16215644

[B30] Laport M. S. Pontes P. V. M. dos Santos D. S. Santos-Gandelman J. d. F. Muricy G. Bauwens M.. (2016). Antibiotic resistance genes detected in the marine sponge *Petromica citrina* from Brazilian coast. Braz. J. Microbiol. 47, 617–620. 10.1016/j.bjm.2016.04.016 27287338 PMC4927650

[B31] Laport M. S. Santos O. C. Muricy G. (2009). Marine Sponges: potential sources of new antimicrobial drugs. Curr. Pharm. Biotechnol. 10, 86–105. 10.2174/138920109787048625 19149592

[B32] Lenski R. E. (1998). Bacterial evolution and the cost of antibiotic resistance. Int. Microbiol. 1, 265–270. 10943373

[B33] Leplae R. Hebrant A. Wodak S. J. Toussaint A. (2004). ACLAME: a CLAssification of Mobile genetic Elements. Nucleic Acids Res. 32(Database issue), D45–D49. 10.1093/nar/gkh084 14681355 PMC308818

[B34] Lutz R. Bujard H. (1997). Independent and tight regulation of transcriptional units in *Escherichia coli* via the LacR/O, the TetR/O and AraC/I1-I2 regulatory elements. Nucleic Acids Res. 25, 1203–1210. 10.1093/nar/25.6.1203 9092630 PMC146584

[B35] Macinga D. R. Cook G. M. Poole R. K. Rather P. N. (1998). Identification and characterization of aarF, a locus required for production of ubiquinone in *Providencia stuartii* and *Escherichia coli* and for expression of 2′-N-acetyltransferase in *P*. stuartii. J. Bacteriol. 180, 128–135. 9422602 10.1128/jb.180.1.128-135.1998 PMC106858

[B36] McArthur A. G. Waglechner N. Nizam F. Yan A. Azad M. A. Baylay A. J.. (2013). The comprehensive antibiotic resistance database. Antimicrob. Agents Chemother. 57, 3348–3357. 10.1128/AAC.00419-13 23650175 PMC3697360

[B37] Mehbub M. F. Lei J. Franco C. Zhang W. (2014). Marine Sponge derived natural products between 2001 and 2010: trends and opportunities for discovery of bioactives. Mar. Drugs 12, 4539–4577. 10.3390/md12084539 25196730 PMC4145330

[B38] Menezes C. B. Bonugli-Santos R. C. Miqueletto P. B. Passarini M. R. Silva C. H. Justo M. R.. (2010). Microbial diversity associated with algae, ascidians and sponges from the north coast of Sao Paulo state, Brazil. Microbiol. Res. 165, 466–482. 10.1016/j.micres.2009.09.005 19879115

[B39] Mitchell A. Chang H. Y. Daugherty L. Fraser M. Hunter S. Lopez R.. (2015). The InterPro protein families database: the classification resource after 15 years. Nucleic Acids Res. 43(Database issue), D213–D221. 10.1093/nar/gku1243 25428371 PMC4383996

[B40] Mori T. Mizuta S. Suenaga H. Miyazaki K. (2008). Metagenomic screening for bleomycin resistance genes. Appl. Environ. Microbiol. 74, 6803–6805. 10.1128/AEM.00873-08 18791008 PMC2576686

[B41] Munck C. Albertsen M. Telke A. Ellabaan M. Nielsen P. H. Sommer M. O. (2015). Limited dissemination of the wastewater treatment plant core resistome. Nat. Commun. 6, 8452. 10.1038/ncomms9452 26419330 PMC4598724

[B42] Muscholl-Silberhorn A. Thiel V. Imhoff J. F. (2008). Abundance and bioactivity of cultured sponge-associated bacteria from the Mediterranean sea. Microb. Ecol. 55, 94–106. 10.1007/s00248-007-9255-9 17497228

[B43] Phelan R. W. Clarke C. Morrissey J. P. Dobson A. D. O'Gara F. Barbosa T. M. (2011). Tetracycline resistance-encoding plasmid from *Bacillus* sp. strain #24, isolated from the marine sponge *Haliclona simulans*. Appl. Environ. Microbiol. 77, 327–329. 10.1128/Aem.01239-10 21057017 PMC3019722

[B44] Reeves A. Z. Campbell P. J. Sultana R. Malik S. Murray M. Plikaytis B. B.. (2013). Aminoglycoside cross-resistance in *Mycobacterium tuberculosis* due to mutations in the 5′ untranslated region of whiB7. Antimicrob. Agents Chemother. 57, 1857–1865. 10.1128/AAC.02191-12 23380727 PMC3623337

[B45] Rodrigues J. V. Bershtein S. Li A. Lozovsky E. R. Hartl D. L. Shakhnovich E. I. (2016). Biophysical principles predict fitness landscapes of drug resistance. Proc. Natl. Acad. Sci. U.S.A. 113, E1470–E1478. 10.1073/pnas.1601441113 26929328 PMC4801265

[B46] Sandegren L. (2014). Selection of antibiotic resistance at very low antibiotic concentrations. Ups. J. Med. Sci. 119, 103–107. 10.3109/03009734.2014.904457 24694026 PMC4034545

[B47] Schupp T. Toupet C. Engel N. Goff S. (1998). Cloning and sequence analysis of the putative rifamycin polyketide synthase gene cluster from *Amycolatopsis mediterranei*. FEMS Microbiol. Lett. 159, 201–207. 10.1111/j.1574-6968.1998.tb12861.x 9503613

[B48] Segawa T. Takeuchi N. Rivera A. Yamada A. Yoshimura Y. Barcaza G.. (2013). Distribution of antibiotic resistance genes in glacier environments. Environ. Microbiol. Rep. 5, 127–134. 10.1111/1758-2229.12011 23757141

[B49] Sengupta S. Chattopadhyay M. K. Grossart H. P. (2013). The multifaceted roles of antibiotics and antibiotic resistance in nature. Front. Microbiol. 4:47. 10.3389/fmicb.2013.00047 23487476 PMC3594987

[B50] Sievers F. Wilm A. Dineen D. Gibson T. J. Karplus K. Li W.. (2011). Fast, scalable generation of high-quality protein multiple sequence alignments using Clustal Omega. Mol. Syst. Biol. 7:539. 10.1038/msb.2011.75 21988835 PMC3261699

[B51] Siguier P. Perochon J. Lestrade L. Mahillon J. Chandler M. (2006). ISfinder: the reference centre for bacterial insertion sequences. Nucleic Acids Res. 34(Database issue), D32–D36. 10.1093/nar/gkj014 16381877 PMC1347377

[B52] Sommer M. O. Dantas G. Church G. M. (2009). Functional characterization of the antibiotic resistance reservoir in the human microflora. Science 325, 1128–1131. 10.1126/science.1176950 19713526 PMC4720503

[B53] Srivastava A. Singhal N. Goel M. Virdi J. S. Kumar M. (2014). CBMAR: a comprehensive beta-lactamase molecular annotation resource. Database (Oxford). 2014:bau111. 10.1093/database/bau111 25475113 PMC4255060

[B54] Summers A. O. (2006). Genetic linkage and horizontal gene transfer, the roots of the antibiotic multi-resistance problem. Anim. Biotechnol. 17, 125–135. 10.1080/10495390600957217 17127524

[B55] Teuber M. Meile L. Schwarz F. (1999). Acquired antibiotic resistance in lactic acid bacteria from food. Antonie Van Leeuwenhoek 76, 115–137. 10.1023/A:1002035622988 10532375

[B56] Untergasser A. Nijveen H. Rao X. Bisseling T. Geurts R. Leunissen J. A. (2007). Primer3Plus, an enhanced web interface to Primer3. Nucleic Acids Res. 35(Web Server issue), W71–W74. 10.1093/nar/gkm306 17485472 PMC1933133

[B57] Versluis D. D'Andrea M. M. Ramiro Garcia J. Leimena M. M. Hugenholtz F. Zhang J.. (2015). Mining microbial metatranscriptomes for expression of antibiotic resistance genes under natural conditions. Sci. Rep. 5:11981. 10.1038/srep11981 26153129 PMC4495384

[B58] Vogel S. (1977). Current-induced flow through living sponges in nature. Proc. Natl. Acad. Sci. U.S.A. 74, 2069–2071. 10.1073/pnas.74.5.2069 266728 PMC431075

[B59] Wagner-Döbler I. Biebl H. (2006). Environmental biology of the marine *Roseobacter* lineage. Annu. Rev. Microbiol. 60, 255–280. 10.1146/annurev.micro.60.080805.142115 16719716

[B60] Walsh F. Duffy B. (2013). The culturable soil antibiotic resistome: a community of multi-drug resistant bacteria. PLoS ONE 8:e65567. 10.1371/journal.pone.0065567 23776501 PMC3680443

[B61] Webster N. S. Taylor M. W. (2012). Marine sponges and their microbial symbionts: love and other relationships. Environ. Microbiol. 14, 335–346. 10.1111/j.1462-2920.2011.02460.x 21443739

[B62] Wichmann F. Udikovic-Kolic N. Andrew S. Handelsman J. (2014). Diverse antibiotic resistance genes in dairy cow manure. mBio 5:e01017–13. 10.1128/mBio.01017-13 24757214 PMC3993861

[B63] Yang J. Wang C. Shu C. Liu L. Geng J. Hu S.. (2013). Marine sediment bacteria harbor antibiotic resistance genes highly similar to those found in human pathogens. Microb. Ecol. 65, 975–981. 10.1007/s00248-013-0187-2 23370726

[B64] Zhang L. An R. Wang J. Sun N. Zhang S. Hu J.. (2005). Exploring novel bioactive compounds from marine microbes. Curr. Opin. Microbiol. 8, 276–281. 10.1016/j.mib.2005.04.008 15939350

[B65] Zhu W. Lomsadze A. Borodovsky M. (2010). Ab initio gene identification in metagenomic sequences. Nucleic Acids Res. 38, e132. 10.1093/nar/gkq275 20403810 PMC2896542

