# Supplementary material for: Sponge Microbiota Are a Reservoir of Functional Antibiotic Resistance Genes
Source: Front Microbiol. 2016 Nov 17;7:1848. doi: 10.3389/fmicb.2016.01848 (PMC5112248; doi:10.3389/fmicb.2016.01848)
Supplement: Supplementary file 1 [file DataSheet1.ZIP › Supplementary Materials and Methods.pdf]

Supplementary Materials and Methods

Methods for the isolation and antibiotic resistance profiling of the 31 bacterial strains

31 bacterial strains were isolated under aerobic conditions from the Mediterranean sponges *A. aerophoba*, *C. candelabrum* and *P. ficiformis* in a previous study (Versluis *et al*, under review). Genomic DNA from these strains was used to prepare small-insert library I-31.

The following five agar media were used to isolate the 31 strains: (I) 37.40 g/l marine broth 2216 (Difco) in Milli-Q; (II) 3.74 g/l marine broth 2216 (Difco) in Milli-Q ; (III) 22 g/l BBL Mueller Hinton broth (BD) in ASW; (IV) 2.2 g/l BBL Mueller Hinton broth (BD) in ASW; (V) 1 g/l porcine stomach mucin (Sigma) in ASW. All media contained 15 g/l noble agar (Sigma-Aldrich). Media III to V were supplemented with 1 ml/l trace metal solution (Olson and McCarthy, 2005), 1 ml/l phosphate solution (Olson and McCarthy, 2005) and 1 ml/l vitamin solution (BME vitamins, Sigma), with the pH adjusted to 7.7 before autoclaving. Solutions of phosphate, vitamins and antibiotics were filter-sterilized and added after autoclaving in order to prevent precipitation or inactivation. Below is tabulated on which medium each strain was isolated.

In the previous study, antibiotic profiling of these strains was done for the following antibiotics: polymyxin B (15 µg/ml), erythromycin (15 µg/ml), ciprofloxacin (15 µg/ml), cefotaxime (100 µg/ml), tetracycline (50 µg/ml), chloramphenicol (50 µg/ml), rifampicin (7.5 µg/ml), ampicillin (50 µg/ml) and imipenem (10 µg/ml).

Table 1 31 bacterial strains were isolated from the sponges *A. aerophoba*, *C. candelabrum* and *P. ficiformis* in a previous study.

Strain ID	Genbank accession of the 16S rRNA gene	Source	Isolated on medium no.	Description of the medium
Bacillus algicola strain DN53_3H5	KP769416	*P. ficiformis*	I	Marine agar
Bacillus idriensis strain DN51_2A1	KP769417	*A. aerophoba*	III	Mueller-Hinton agar
Brachybacterium paraconglomeratum strain DN73_5E10	KP769418	*A. aerophoba*	V	Mucin agar
Brevibacterium sp. DN213_3F7	KP769419	*A. aerophoba*	III	Mueller-Hinton agar
Microbulbifer sp. DN217_4H2	KP769420	*C. candelabrum*	I	Marine agar
Ruegeria atlantica strain DN12_1A11	KP769421	*A. aerophoba*	I	Marine agar
Psychrobacter celer strain DN193_4B9	KP769422	*P. ficiformis*	III	Marine agar (10X diluted nutrients)
Janibacter melonis strain DN216_4B10	KP769423	*A. aerophoba*	IV	Mueller-Hinton agar (10X diluted nutrients)
Pseudovibrio ascidiaceicola strain DN64_8G1	KP769424	*P. ficiformis*	V	Mucin agar
Pseudovibrio ascidiaceicola strain DN64_1D03	KP769425	*A. aerophoba*	I	Marine agar
Rhodococcus jialingiae strain DN106_7C1	KP769426	*A. aerophoba*	V	Mucin agar
Acinetobacter radioresistens strain DN138_5C8	KP769427	*A. aerophoba*	III	Marine agar (10X diluted nutrients)
Pseudovibrio sp. DN49_8H4	KP769428	*P. ficiformis*	IV	Mueller-Hinton agar (10X diluted nutrients)
Nonlabens arenilitoris strain DN166_3E9	KP769429	*A. aerophoba*	I	Marine agar
Leisingera aquimarina strain DN172_5F6	KP769430	*A. aerophoba*	V	Mucin agar
Flavobacteriaceae sp. DN105_1H3	KP769431	*A. aerophoba*	I	Marine agar
Ruegeria atlantica strain DN83_2B6	KP769432	*A. aerophoba*	I	Marine agar
Pseudomonas oryzihabitans strain DN90_5E11	KP769433	*C.candelabrum*	II	Marine agar (10X diluted nutrients)
Ruegeria sp. DN110_6H4	KP769434	*A. aerophoba*	II	Marine agar (10X diluted nutrients)
Bacillus sp. DN88_4G3	KP769435	*P. ficiformis*	III	Mueller-Hinton agar
Bacillus aryabhattai strain DN67_5C7	KP769436	*A. aerophoba*	II	Marine agar (10X diluted nutrients)
Aquimarina megaterium strain DN30_1H2	KP769437	*A. aerophoba*	I	Marine agar
Sphingomonas sp. DN81_6F7	KP769438	*P. ficiformis*	III	Mueller-Hinton agar
Ruegeria sp. DN71_7G3	KP769439	*A. aerophoba*	IV	Mueller-Hinton agar (10X diluted nutrients)
Bacillus horikoshii strain DN9_1A9	KP769440	*A. aerophoba*	I	Marine agar
Flavobacteriaceae sp. DN50_6C1	KP769441	*A. aerophoba*	V	Mucin agar
Mycobacterium peregrinum strain DN74_7A10	KP769442	*P. ficiformis*	I	Marine agar
Bradyrhizobium pachyrhizi strain DN55_6A7	KP769443	*A. aerophoba*	II	Marine agar (10X diluted nutrients)
Flavobacteriaceae sp. DN112_6A5	KP769444	*A. aerophoba*	V	Mucin agar
Pseudovibrio sp. DN206_4B7	KP769445	*P. ficiformis*	IV	Mueller-Hinton agar (10X diluted nutrients)
Bacillus stratosphericus strain DN14_7A9	KP769446	*P. ficiformis*	IV	Mueller-Hinton agar (10X diluted nutrients)

Olson, J.B., and McCarthy, P.J. (2005). Associated bacterial communities of two deep-water sponges. *Aquatic Microbial Ecology* 39(1), 47-55. doi: 10.3354/Ame039047.
