# Supplementary material for: Sponge Microbiota Are a Reservoir of Functional Antibiotic Resistance Genes
Source: Front Microbiol. 2016 Nov 17;7:1848. doi: 10.3389/fmicb.2016.01848 (PMC5112248; doi:10.3389/fmicb.2016.01848)
Supplement: Supplementary file 1 [file DataSheet1.ZIP › Supplementary Tables S1-S3_larger_fonts.pdf]

Supplementary Tables S1-S3

Functional metagenomics confirm sponge microbiota as a reservoir of antibiotic resistance genes

Dennis Versluis[1], Mari Rodriguez de Evgrafov[2], Morten O.A. Sommer[2], Detmer Sipkema[1], Hauke Smidt[1], Mark W.J. van Passel[1,3]

[1]Laboratory of Microbiology, Wageningen University, Wageningen, The Netherlands

[2]Novo Nordisk Foundation Center for Biosustainability, Technical University of Denmark, Hørsholm, Denmark

[3]National Institute for Public Health and the Environment, Bilthoven, The Netherlands

Supplementary Table S1. Resistance genes that were identified on the inserts meeting our threshold (E-value < 1.0E-7) using either the CARD or Resfams database as a reference. The best hit is shown of BLASTp of the resistance gene against NCBI's non-redundant protein database. Based on the sequences of both the resistance gene and the aforementioned best hit a global alignment percentage was calculated by Clustal Omega. Gene-specific PCRs with custom-designed primers were used to assign the genes to the bacterium of origin. The sequences of the custom-designed primers are given as well as the position at which they bind.

							Gene-specific PCRs				BLASTp against NCBI's non-redundant protein sequences							Resfams domain search					BLASTp against CARD					BLASTn against CARD				
Clone accession	Gene	Library	Resistance phenotype	Clone ID	Bacterium to which the AR gene was assigned	Strand	FW primer position of 5' end	Sequences	REV primer position of the 3' end	Sequence	Accession best hit	Annotation	E-value	%id	Bitscore	Aln_length	% identity after global alignment by ClustalW	Resfam ID	E-value	Mechanism classification	β-lactamase Ambler class	Description	Best hit	% identity	Alignment length	E-value	Bitscore	Best hit	% identity	Alignment length	E-value	Bitscore
KU577908	42..860	I-31	amp	Iso_Amp1	*Bacillus stratosphericus* DN14_7A9	+	410	GGACCATACACTGGGCTCTG	672	TCATTCGCTTGACGGTTCTC	EMI12369.1	beta-lactamase [Bacillus stratosphericus LAMA 585]	0.0	97	529	252	93.01	RF0056	1.2E-78	Beta-Lactamase	Class D	Class D beta-lactamases [ARO:3000075;ARO:3000017]	JX893517.1.gene1.p01 beta-lactamase OXA-247	37.76	241	1E-52	172	KF203107.1.gene1 blaOXA-333	88.57	35	2E-05	46.4
KU577909	284..1,216	I-31	amp	Iso_Amp2	*Bacillus aryabhattai* DN67_5C7	+	369	GCTTTTCAAGCAGCGAGGTA	1,118	GGATCGCAAGAACAATTGGA	WP_013058055.1	MULTISPECIES: class A beta-lactamase [Bacillus]	0.0	100	640	310	100	RF0053	2.5E-98	Beta-Lactamase	Class A	Class A beta-lactamase [ARO:3000078]	AY453161.1.gene1.p01 beta-lactamase I	54.18	299	5E-121	352	AY453161.1.gene1 bla1	66.53	747	1E-53	206
KU577910	880..1,692	I-31	amp	Iso_Amp3	*Pseudovibrio ascidiaceicola* DN64_8G1 & DN64_1D03	+	1,141	GTTGATCGCGGTGAAGAGAG	1,649	TTTCCGGCCAGATAACACTG	WP_057463840.1	class A beta-lactamase [Pseudovibrio sp. POLY-S9]	1E-152	96	440	218	85.33	RF0053	1E-84	Beta-Lactamase	Class A	Class A beta-lactamase [ARO:3000078]	AM850912.2.gene1.p01 beta-lactamase	54.02	224	3E-75	231	DQ174304.1.gene1 blaSHV-64	81.13	53	6E-07	51.8
KU577911	654..1,154	I-31	chlor	Iso_Chlor1	*Bacillus stratosphericus* DN14_7A9	+	146	TAATCCGGACGGAATTCACA	1,360	CCCTTATTCCCCTTCCACTG	WP_041507443.1	chloramphenicol acetyltransferase CAT [Bacillus aerophilus]	4E-119	100	348	166	100	RF0045	1.1E-70	Acetyltransferase	-	PF00302.13 Chloramphenicol acetyltransferase [ARO:3000122]	K00544.gene.p01 cat-86* protein	93.37	166	4E-116	328	K00544.gene cat-86	93.41	501	0.0	755
KU577912	2,941..3,270	I-31	gent	Iso_Gent1	*Bacillus stratosphericus* DN14_7A9	+	2,900	AAGCGGACTTGGGGTGAA	3,088	GGTTCCTTGCAAACCGAAA	WP_010096956.1	aminoglycoside 2'-O-phosphotransferase [Ornithinibacillus scapharcae]	1E-22	54	99.4	51	47.71						U51479.gene.p01 APH2-Ic protein	37.96	108	2E-22	86.7	JX131372.1.gene1 blaIMP-37	100.00	15	2.5	28.3
KU577912	3,399..3,845	I-31	gent	Iso_Gent1	*Bacillus stratosphericus* DN14_7A9	+	3,455	TATTGCCACACGGTTTGATG	3,696	GGGCGTGACCTCTTACCTTC	WP_046503909.1	hypothetical protein [Paenibacillus riograndensis]	1E-43	51	157	73	49.66	RF0032	3E-13	Acetyltransferase	-	PF01636.18 Phosphotransferase enzyme family [ARO:3000000]	U51479.gene.p01 APH2-Ic protein	48.48	132	5E-42	141	U51479.gene APH2-Ic	70.06	157	4E-12	68.0
KU577919	884..2,065	I-31	ami	Iso_Ami1	*Bacillus horikoshii* DN9_1A9	-	890	TGTTGCTTCCATACCCTTCAA	1,874	CGACGTGGAAATCGTGAAAT	WP_010198793.1	aminoglycoside phosphotransferase APH(3') [Bacillus sp. m3-13]	7E-137	80	404	191	77.96	RF0032	1.4E-24	Acetyltransferase	-	PF01636.18 Phosphotransferase enzyme family [ARO:3000000]	X90856.1.gene1.p01 aminoglycoside phosphotransferase (3')Iips	27.59	174	4E-10	56.6	L20573.1.gene1 bcrA	100.00	17	0.78	31.9
KU577924	71..631	I-31	trim	Iso_Trim1	*Flavobacteriaceae* sp. DN50_6C1	+	258	TGGGCAGAAAGACCTTTGAA	787	TGGGCAGAAAGACCTTTGAA	WP_026813745.1	hypothetical protein [Arenibacter certesii]	6E-77	70	239	111	68.1	RF0063	5.6E-58	Antibiotic Target	-	PF00186.14 Dihydrofolate reductase [ARO:3000745]	AJ605332.1.gene1.p01 dihydrofolate reductase	40.70	172	5E-39	130	KF460533.1.gene1 blaOXA-363	100.00	16	1.3	30.1
KU577925	1..648	I-31	trim	Iso_Trim2	*Nonlabens arenilitoris* DN166_3E9	+	29	CAGGATGGGAGAAACGTCAG	1,034	CAGTGAATGACCTGCAACCA	EAS20419.1	dihydrofolate reductase [Flavobacteria bacterium BBFL7]	4E-154	99	439	214	99.53	RF0063	3.9E-62	Antibiotic Target	-	PF00186.14 Dihydrofolate reductase [ARO:3000745]	J03306.gene.p01 dfrA3 protein	41.51	159	2E-43	143	DQ267940.1.gene1 dfrA25	100.00	17	0.42	31.9
KU577926	3,032..3,562	I-31	trim	Iso_Trim3	*Pseudovibrio* sp. DN49_8H4	+	2,858	AAACTGCCGCTTATGGTGTG	3,232	CACGATATTGGTTCGGTTGG	WP_057465752.1	diacylglycerol kinase [Pseudovibrio sp. POLY-S9]	3E-95	78	286	134	77.46	RF0063	1.7E-58	Antibiotic Target	-	PF00186.14 Dihydrofolate reductase [ARO:3000745]	AM403715.1.gene1.p01 putative trimethoprim resistance dihydrofolate reductase	43.79	169	1E-50	161	FM957884.1.gene1 dfrA22	92.86	28	2E-4	42.8
KU577927	254..736	I-31	trim	Iso_Trim4	*Bacillus horikoshii* DN9_1A9	+	324	TACCTGCCGATTTGGCATAC	630	GGAAAGTACGTATCCCCATCG	WP_010193820.1	dihydrofolate reductase [Bacillus sp. m3-13]	2E-89	80	270	127	79.38	RF0063	1.4E-64	Antibiotic Target	-	PF00186.14 Dihydrofolate reductase [ARO:3000745]	U43152.1.gene1.p01 dihydrofolate reductase DHFR L1 protein	51.25	160	5E-58	179	AB205645.1.gene2 dfrG	64.59	466	1E-12	69.8
KU577928	1,639..2,124	I-31	trim	Iso_Trim5	*Bacillus algicola* DN53_3H5	+	836	GGAGGGAAAGCATGAAAACA	2,075	TCTTTCGTATGTTGATCTCTCGACT	WP_048309627.1	dihydrofolate reductase [Anaerobacillus macyae]	1E-77	69	240	111	69.38	RF0063	7.3E-67	Antibiotic Target	-	PF00186.14 Dihydrofolate reductase [ARO:3000745]	J03306.gene.p01 dfrA3 protein	45.96	161	4E-50	158	EF219134.3.gene12 unnamed gene.	84.78	46	3E-07	51.8
KU577929	1,160..1,645	I-31	trim	Iso_Trim6	*Bacillus* sp. DN88_4G3	-	1,263	GGGAAATACGTGTCACCTTCG	1,583	TTGGAGATTGCCTGCTGATT	WP_018707221.1	hypothetical protein [Bacillus fordii]	4E-88	76	266	122	75.78	RF0063	1.1E-66	Antibiotic Target	-	PF00186.14 Dihydrofolate reductase [ARO:3000745]	FN812951.1.gene1.p01 dihydrofolate reductase	50.00	160	4E-58	179	FN812951.1.gene1 dfrK	70.44	159	3E-14	75.2
KU577930	1,576..2,088	I-31	trim	Iso_Trim7	*Acinetobacter radioresistens* DN138_5C8	+	1,563	CACCGGTGGCTGTATGAGTT	1,953	AAGCTCCAGACGGTCAGCTA	WP_005023315.1	MULTISPECIES: diacylglycerol kinase [Acinetobacter]	4E-122	100	353	170	100	RF0063	1.9E-55	Antibiotic Target	-	PF00186.14 Dihydrofolate reductase [ARO:3000745]	J03306.gene.p01 dfrA3 protein	40.74	162	7E-39	130	AF512546.1.gene1 dfrV	79.07	43	0.027	35.6
KU577931	623..841	I-31	trim	Iso_Trim8	*Aquimarina megaterium* DN30_1H2	-	631	TGATGTACGTCAAATACGTAAAAGGA	796	GGGATGGATCATGCAGATTG	WP_025665158.1	dihydrofolate reductase [Aquimarina megaterium]	1E-41	100	148	70	97.22	RF0063	5.1E-22	Antibiotic Target	-	PF00186.14 Dihydrofolate reductase [ARO:3000745]	AM403715.1.gene1.p01 putative trimethoprim resistance dihydrofolate reductase	35.82	67	3E-13	58.5	AF148067.1.gene1 tla-1	90.00	20	1.6	28.3
KU577931	810..1,562	I-31	trim	Iso_Trim8	*Aquimarina megaterium* DN30_1H2	-	1,028	CATCTGGTAAATGCCAAACCA	1,699	TCCCAGAAGATGCTGTATTGAGA	WP_025665158.1	dihydrofolate reductase [Aquimarina megaterium]	2E-174	99	495	242	96.8	RF0063	4E-35	Antibiotic Target	-	PF00186.14 Dihydrofolate reductase [ARO:3000745]	AJ605332.1.gene1.p01 dihydrofolate reductase	42.11	95	8E-24	92.8	FN812951.1.gene1 dfrK	80.00	45	0.14	33.7
KU577932	4,902..5,537	I-31	trim	Iso_Trim10	*Flavobacteriaceae* sp. DN112_6A5	+	4,904	GGGAAAAGAATGGGAAAAAGC	5,459	TTTCCAAGTACTTTCATCTATCTCAGG	WP_042502644.1	dihydrofolate reductase [Algibacter lectus]	4E-109	72	328	156	73.93	RF0063	2.6E-64	Antibiotic Target	-	PF00186.14 Dihydrofolate reductase [ARO:3000745]	J03306.gene.p01 dfrA3 protein	39.62	159	2E-39	133	KF534911.1.gene1 dfrA15	95.45	22	0.034	35.6
KU577933	1,718..2,173	I-31	trim	Iso_Trim11	*Psychrobacter celer* DN193_4B9	+	1,527	CCGCGACTTTCAAAGCAAAAGTCGCGGTATTGCTTTGATA	1,918	TTGCGTTAAAGCATCATCCA	WP_025651502.1	MULTISPECIES: dihydrofolate reductase [Psychrobacter]	1E-107	80	319	155	80	RF0063	6.5E-38	Antibiotic Target	-	PF00186.14 Dihydrofolate reductase [ARO:3000745]	AM403715.1.gene1.p01 putative trimethoprim resistance dihydrofolate reductase	30.11	186	6E-20	81.3	EF219134.3.gene12 unnamed gene	84.62	39	5E-5	44.6
KU577934	454..939	I-31	trim	Iso_Trim12	*Flavobacteriaceae* sp. DN105_1H3	-	564	ACGTATCCGCCTCGAAATCT	972	CAAGGAATCTTGGAACAGAACAA	WP_034043103.1	dihydrofolate reductase [Flaviramulus ichthyoenteri]	1E-92	82	283	130	80.75	RF0063	8.1E-64	Antibiotic Target	-	PF00186.14 Dihydrofolate reductase [ARO:3000745]	AM403715.1.gene1.p01 putative trimethoprim resistance dihydrofolate reductase	44.24	165	8E-42	137	KJ174469.1.gene1 dfrA8	72.58	62	0.026	35.6
KU577935	425..841	I-31	rif	Iso_Rif1	*Bacillus stratosphericus* DN14_7A9	-	122	CTCACCCGCTCATCACAAAT	822	TTTTAGATCCTGGGCCGTTT	WP_046342521.1	MULTISPECIES: ribosomal subunit interface protein [Bacillus]	2E-96	99	286	137	99.28						AF001493.1.orf0.gene.p01 rifampin ADPribosyl transferase	54.48	134	5E-48	151	AF078527.1.gene5 arr-2	76.54	81	5E-10	60.8
KU577936	5,293..5,709	I-31	rif	Iso_Rif2	*Bacillus aryabhattai* DN67_5C7	+	5,444	CTGCTAAATGGGGTGCTGAA	5,773	GCCTTCATTGTAGGTATGCTATATGTT	WP_012209781.1	ribosomal subunit interface protein [Bacillus megaterium]	2E-95	100	283	138	100						AF001493.1.orf0.gene.p01 rifampin ADPribosyl transferase	54.14	133	1E-46	147	AF078527.1.gene5 arr-2	77.78	63	3E-7	51.8
KU577938	139..531	Pf	ami	Env_Ami1	n/a	+	n/a	n/a	n/a	n/a	WP_040343406.1	GNAT family acetyltransferase [Bacillus nealsonii]	2E-26	39	107	45	35.94	RF0014	5.5E-15	Acetyltransferase	-	PF13508.1 Acetyltransferase (GNAT) domain [ARO:3000000]	L12710.gene.p01 AAC6-Ii protein	30.63	111	1E-4	36.6	KF952266.1.gene2 aadA5	94.44	18	3.0	28.3
KU577940	199..711	Pf	trim	Env_Trim1	n/a	+	n/a	n/a	n/a	n/a	KKQ67813.1	Dihydrofolate reductase [Parcubacteria bacterium GW2011_GWA2_38_27]	2E-54	51	181	84	51.85	RF0063	2.5E-58	Antibiotic Target	-	PF00186.14 Dihydrofolate reductase [ARO:3000745]	J03306.gene.p01 dfrA3 protein	46.34	164	1E-47	152	AB205645.1.gene2 dfrG	83.78	37	6E-4	41.0
KU577941	176..664	Pf	trim	Env_Trim2	n/a	+	n/a	n/a	n/a	n/a	KKQ67813.1	Dihydrofolate reductase [Parcubacteria bacterium GW2011_GWA2_38_27]	8E-56	48	187	78	48.15	RF0063	7.9E-59	Antibiotic Target	-	PF00186.14 Dihydrofolate reductase [ARO:3000745]	FN812951.1.gene1.p01 dihydrofolate reductase	45.68	162	8E-53	167	Z50802.3.gene1 dfrxiii	67.26	113	7E-3	37.4
KU577942	398..889	Pf	trim	Env_Trim3	n/a	+	n/a	n/a	n/a	n/a	KKQ21453.1	dihydrofolate reductase [Parcubacteria bacterium GW2011_GWA2_37_10]	1E-51	51	174	83	51.27	RF0063	7.2E-58	Antibiotic Target	-	PF00186.14 Dihydrofolate reductase [ARO:3000745]	FN812951.1.gene1.p01 dihydrofolate reductase	42.94	163	2E-44	144	J03306.gene dfrA3	72.09	86	4E-6	48.2
KU577944	2,700..3,374	Aa	ami	Env_Ami2	n/a	-	n/a	n/a	n/a	n/a	WP_051294704.1	glycerol-3-phosphate acyltransferase [Gemmobacter nectariphilus]	2E-13	33	79.0	74	31.98	RF0018	3.4E-07	Acetyltransferase	-	PF01553.16 Acyltransferase [ARO:3000000]	FN594949.1.gene24.p01 ribosomal tetracycline resistance protein tet(44)	29.73	37	5.6	24.6	AF079138.1.gene1 pikR2	85.71	28	0.13	33.7

Supplementary Table S2. Inserts that confer resistance against amikacyn, D-cycloserine or trimethoprim on which no AR gene could be identified with high confidence. We defined a resistance gene to be identified with high confidence if it was detected at an e-value of <1E-7 by either BLASTp or BLASTn against the CARD database, or by employing the pHMMs of the Resfams database. The best hit is shown of BLASTp of the genes against NCBI's non-redundant protein database. Based on the sequences of both the gene and the aforementioned best hit a global alignment percentage was calculated by Clustal Omega. The translated sequences of the genes were analysed by InterProScan. Gene-specific PCRs with custom-designed primers were used to assign the inserts to the bacterium of origin. The sequences of the custom-designed primers are given as well as the position at which they bind.

									Interproscan results				Insert-specific PCRs				
**Clone ID**	**Clone accession**	**Library**	**Resistance phenotype**	**Gene**	**Best hit NCBI (nr/nt)**	**Annotation**	**% amino acid identity**	**Predicted resistance function**	**Protein family membership**	**Biolocial process**	**Molecular function**	**Cullular component**	**Assigned to**	**FW detection primer position of 5' end**	**Sequences**	**REV detection primer position of the 3' end**	**Sequence**
Iso_Ami2	KU577920	I-31	ami	2..934	WP_039537676.1	Flagellar motor protein [Ruegeria sp. ANG-R]	92.3	Aminoglycoside 6'-N-acetyltransferase	None predicted	None predicted	None predicted	None predicted	*Bacillus stratosphericus* DN14_7A9	3,285	TAAAGCGGATGCATCAGGAG	3,669	CATCCCAGCAAATGCTTCAT
				1,043..1,687	WP_052261261.1	Hypothetical protein [Ruegeria sp. ANG-S4]	57.9		None predicted	None predicted	None predicted	None predicted					
				1,808..2,197	CUJ84255.1	ATP-dependent Clp protease ATP-binding subunit ClpA [Ruegeria sp. CECT 5091]	98.5		None predicted	None predicted	None predicted	None predicted					
				2,293..3,741	WP_045033790.1	MULTISPECIES: copper amine oxidase [Bacillus]	99.6		None predicted	None predicted	None predicted	None predicted					
Iso_Ami3	KU577921	I-31	ami	2..526	WP_057464053.1	16S rRNA (guanine(966)-N(2))-methyltransferase RsmD [Pseudovibrio sp. POLY-S9]	98.9	Aminoglycoside methyltransferase	RNA methyltransferase, RsmD (IPR004398)	GO:0031167 rRNA methylation, GO:0032259 methylation	GO:0003676 nucleic acid binding, GO:0008168 methyltransferase activity	None predicted	*Pseudovibrio ascidiaceicola* DN64_1D03	106	ACGCGTTCTGGACCTGTTT	524	ACACGCCGAGATACCTGAGA
				1,661..2,977	WP_021815359.1	Transporter [Psychrobacter aquaticus]	86.1		Drug resistance transporter EmrB/QacA subfamily (IPR004638), Major facilitator superfamily (IPR011701)	GO:0006810 transport, GO:0055085 transmembrane transport	None predicted	GO:0016021 integral component of membrane	*Pseudovibrio ascidiaceicola* DN64_8G1	106	ACGCGTTCTGGACCTGTTT	524	ACACGCCGAGATACCTGAGA
Iso_Ami4	KU577922	I-31	ami	1..1,329	WP_014283807.1	Amino acid transporter [Pseudovibrio sp. FO-BEG1]	92.7	transmembrane export	Tryptophan/tyrosine permease (IPR018227)	GO:0003333 amino acid transmembrane transport, GO:0006865 amino acid transport	None predicted	GO:0016020 membrane	*Pseudovibrio* sp. DN49_8H4	134	GTGTTATCGCTGCGGGTACT	1,434	GCTGGGTTCCGCAGTAAGTA
Env_Ami3	KU577939	Pf	ami	2..73	No significant hit				None predicted	None predicted	None predicted	None predicted	n/a	1,141	GTTGATCGCGGTGAAGAGAG	1,649	TTTCCGGCCAGATAACACTG
				388..1,005	WP_052888341.1	Non-canonical purine NTP pyrophosphatase [Thermogemmatispora carboxidivorans]	49.1	Aminoglycoside modification	Inosine triphosphate pyrophosphatase-like (IPR029001)	GO:0009143 nucleoside triphosphate catabolic process	GO:0016787 hydrolase activity, GO:0017111 nucleoside-triphosphatase activity	None predicted					
				1,086..1,382	WP_010886911.1	Radical SAM protein [Deinococcus radiodurans]	59.6		None predicted	None predicted	GO:0003824 catalytic activity, GO:0051536 iron-sulfur cluster binding	None predicted					
Iso_Dcy1	KU577913	I-31	D-cycl	1..543	WP_047947080.1	GntR family transcriptional regulator [Bacillus altitudinis]	99.5		None predicted	GO:0006355 regulation of transcription, DNA-templated	GO:0003700 transcription factor activity, sequence-specific DNA binding	None predicted	*Bacillus stratosphericus* DN14_7A9	159	GATGGCGGAAATCAATCTCA	1,871	TCCATTTCACTTTTGCCTCTC
				593..910	EMI13874.1	Major facilitator superfamily transporter [Bacillus stratosphericus LAMA 585]	94.3	antibiotic efflux	None predicted	None predicted	None predicted	None predicted					
				903..1,760	KRV43460.1	MFS transporter [Bacillus sp. TH007]	95.8	antibiotic efflux	Major facilitator superfamily (IPR011701)	GO:0055085 transmembrane transport	None predicted	GO:0016021 integral component of membrane					
				1,928..2,206	WP_041507495.1	Barnase inhibitor [Bacillus aerophilus]	15.9		None predicted	None predicted	None predicted	None predicted					
				2,363..2,785	WP_017366964.1	MULTISPECIES: iron ABC transporter permease [Bacillus]	100		ABC transporter, permease protein (IPR000522)	None predicted	GO:0005215 transporter activity	GO:0016020 membrane					
Iso_Dcy2	KU577914	I-31	D-cycl	,	WP_034639392.1	N-acetylmuramic acid 6-phosphate etherase [Bacillus manliponensis]	72.7		None predicted	None predicted	None predicted	None predicted	*Bacillus* sp. DN88_4G3	278	TCGCCCTATCTGCTTTTCAG	2,163	ACCTCTTCGATCACCCCACT
				333..515	WP_018707511.1	Hypothetical protein [Bacillus fordii]	73.7		None predicted	None predicted	None predicted	None predicted					
				618..1,838	WP_018707512.1	Hypothetical protein [Bacillus fordii]	55.6	antibiotic efflux	Major facilitator superfamily (IPR011701)	GO:0055085 transmembrane transport	None predicted	GO:0016021 integral component of membrane					
				1,955..2,323	WP_047150337.1	NADPH:quinone oxidoreductase [Aneurinibacillus tyrosinisolvens]	74.0		Alcohol dehydrogenase superfamily, zinc-type (IPR002085)	GO:0055114 oxidation-reduction process	GO:0008270 zinc ion binding, GO:0016491 oxidoreductase activity	None predicted					
Iso_Dcy3	KU577915	I-31	D-cycl	3..1,214	WP_053487427.1	GntR family transcriptional regulator [Bacillus sp. FJAT-21351]	100		None predicted	GO:0006355 regulation of transcription, DNA-templated, GO:0009058 biosynthetic process	GO:0003700 transcription factor activity, sequence-specific DNA binding, GO:0003824 catalytic activity , GO:0030170 pyridoxal phosphate binding	None predicted	*Bacillus aryabhattai* DN67_5C7	118	TGTGTATGAGCGGGTAGCAGT	2,346	AGCCTGCTGATTGCTAATGG
				1,365..1,586	AIA13555.1	EamA-like transporter family, partial [uncultured bacterium]	82.2	antibiotic efflux	None predicted	None predicted	None predicted	None predicted					
				1,637..2,284	WP_013057670.1	MULTISPECIES: multidrug transporter [Bacillus]	100		None predicted	None predicted	None predicted	GO:0016020 membrane, GO:0016021 integral component of membrane					
Iso_Dcy4	KU577916	I-31	D-cycl	437..847	WP_041816932.1	Transporter [Bacillus megaterium]	93.4	antibiotic efflux	None predicted	None predicted	None predicted	None predicted	*Bacillus aryabhattai* DN67_5C7	150	TTGCAAAGTGTTAAAGGAATTGA	2,957	TGTGAACCCTGCATATCCAAC
				916..1,596	WP_041816932.1	Transporter [Bacillus megaterium]	99.5	antibiotic efflux	Major facilitator superfamily (IPR011701)	GO:0055085 transmembrane transport	None predicted	GO:0016021 integral component of membrane					
				2,031..3,158	WP_013059985.1	MFS transporter [Bacillus megaterium]	99.7	antibiotic efflux	Major facilitator superfamily (IPR011701)	GO:0055085 transmembrane transport	None predicted	GO:0016021 integral component of membrane					
Iso_Dcy5	KU577917	I-31	D-cycl	15..893	WP_057464804.1	Transporter [Pseudovibrio sp. POLY-S9]	99.3	antibiotic efflux	None predicted	None predicted	None predicted	GO:0016020 membrane, GO:0016021 integral component of membrane	*Pseudovibrio ascidiaceicola* DN64_1D03	121	CCGGCTTTGATCCATTCTTT	1,921	ATGGTGTACCCGTCATCGAA
				944..1,216	WP_057464747.1	Succinate dehydrogenase [Pseudovibrio sp. POLY-S9]	98.9		None predicted	None predicted	None predicted	None predicted	*Pseudovibrio ascidiaceicola* DN64_8G1	121	CCGGCTTTGATCCATTCTTT	1,921	ATGGTGTACCCGTCATCGAA
				1,203..1,946	WP_057464747.1	Succinate dehydrogenase [Pseudovibrio sp. POLY-S9]	91.9		Succinylglutamate desuccinylase/aspartoacylase (IPR007036)	GO:0008152 metabolic process	GO:0016788 hydrolase activity, acting on ester bonds	None predicted					
Iso_Dcy6	KU577918	I-31	D-cycl	1..90	WP_003944391.1	MULTISPECIES: hypothetical protein [Rhodococcus]	100		None predicted	None predicted	None predicted	None predicted	*Ruegeria* sp. DN71_7G3	513	GCAGCAGTTCCTGACCATTC	1,372	CGGGACGGTACAGACGTAAA
				95..259	WP_007726759.1	MULTISPECIES: hypothetical protein [Rhodococcus]	69.9		None predicted	None predicted	None predicted	None predicted	*Ruegeria* sp. DN110_6H4	513	GCAGCAGTTCCTGACCATTC	1,372	CGGGACGGTACAGACGTAAA
				409..924	WP_008758706.1	RTX toxin [Rhodobacteraceae bacterium KLH11]	70.9		RTX secretion protein D, Gram-negative bacteria (IPR003997)	GO:0009306 protein secretion	None predicted	GO:0016020 membrane	*Acinetobacter radioresistens* DN138_5C8	2,240	AAAGGTCATCAGGGCTGCTT	2,851	CTGCAAACTAACTGGGGGTTT
				926..1576	WP_010441452.1	RTX toxin [Ruegeria conchae]	78.7		None predicted	None predicted	None predicted	None predicted					
				1,752..2,105	EGE55388.1	Hypothetical protein RHECNPAF_930033 [Rhizobium etli CNPAF512]	28.2		None predicted	None predicted	None predicted	None predicted					
				2,102..2,362	WP_034675693.1	Membrane protein [Acinetobacter baumannii]	100		Benzoate transporter (IPR004711)	GO:0042919 benzoate transport	GO:0042925 benzoate transporter activity	GO:0016021 integral component of membrane					
				2,467..3,309	GAB74864.1	Putative benzoate transporter [Acinetobacter radioresistens DSM 69	99.6	antibiotic efflux	Benzoate transporter (IPR004711)	GO:0042919 benzoate transport	GO:0042925 benzoate transporter activity	GO:0016021 integral component of membrane					
Iso_Trim13	KU577923	I-31	trim	3..176	WP_050604224.1	Sodium:proton exchanger [Ruegeria sp. 6PALISEP08]	87.7		None predicted	None predicted	None predicted	None predicted	*Ruegeria atlantica* DN83_2B6	381	GACGAAGGGCAGGTCACAT	978	CCTATTCAACGCCCAGAACA
				246..977	WP_037316481.1	Short-chain dehydrogenase [Ruegeria halocynthiae]	84.9		Glucose/ribitol dehydrogenase (IPR002347)	GO:0008152 metabolic process	GO:0016491 oxidoreductase activity	None predicted					
				1,268..3,073	CUK00006.1	Excinuclease ABC subunit C [Ruegeria sp. CECT 5091]	93.3		UvrABC system, subunit C (IPR004791)	GO:0006281 DNA repair	GO:0005515 protein binding, GO:0009381 excinuclease ABC activity	GO:0005737 cytoplasm, GO:0009380 excinuclease repair complex					
				3,143..3,685	WP_037316487.1	Membrane protein [Ruegeria halocynthiae]	74.4		None predicted	None predicted	None predicted	GO:0016020 membrane, GO:0016021 integral component of membrane					
				3,986..4,492	WP_020412004.1	Hypothetical protein [Microbulbifer variabilis]	91.1		Bacterial lipid A biosynthesis acyltransferase (IPR004960)	None predicted	GO:0016740 transferase activity	GO:0016021 integral component of membrane					
Iso_Trim14	KU577937	I-31	trim	3..440	WP_055442403.1	Nitrous-oxide reductase [Lacinutrix himadriensis]	83.5		None predicted	None predicted	GO:0005515 protein binding	None predicted	*Flavobacteriaceae* sp. DN105_1H3	151	CGGCGAAATACGCACATAAT	2,230	AGCATTACAGCCAGCCAAGA
				379..828	WP_027137829.1	Nitrous-oxide reductase [Gaetbulibacter saemankumensis]	85.1		None predicted	None predicted	GO:0005515 protein binding	None predicted					
				800..1,279	WP_027137829.1	Nitrous-oxide reductase [Gaetbulibacter saemankumensis]	83.2		None predicted	None predicted	GO:0005507 copper ion binding	None predicted					
				1,858..2,130	WP_034042796.1	Membrane protein [Flaviramulus ichthyoenteri]	80.2		None predicted	None predicted	None predicted	None predicted					
				2,096..2,410	WP_027879511.1	Hypothetical protein [Mesoflavibacter zeaxanthinifaciens]	77.8		None predicted	None predicted	None predicted	None predicted					
Env_Trim4	KU577943	Aa	trim	3..521	YP_004324049.1	Td thymidylate synthetase [Synechococcus phage Syn19]	80.8		Thymidylate synthase ThyX (IPR003669)	GO:0006231 dTMP biosynthetic process	GO:0050660 flavin adenine dinucleotide binding, GO:0050797 thymidylate synthase (FAD) activity	None predicted	n/a	146	TAATCCGGACGGAATTCACA	1,360	CCCTTATTCCCCTTCCACTG
				586..882	YP_004324369.1	Hypothetical protein SSSM7_321 [Synechococcus phage S-SSM7]	75.3		None predicted	None predicted	None predicted	None predicted					
				884..1,273	BAR30472.1	P-starvation inducible protein [uncultured Mediterranean phage uvMED]	81.5		None predicted	None predicted	GO:0005524 ATP binding	None predicted					

Supplementary Table S3. BLASTn results of resistance genes identified meeting our confidence threshold against the NCBI nr/nt database. Only hits with nucleotide idenity values >90% are shown.

							Hits with >90% nucleotide idenity in NCBI nr/nt			
**Clone ID**	**Clone accession**	**Library**	**Resistance phenotype**	**Gene**	**Bacterium to which the AR gene was assigned**	**Strand**	**Accession**	**Nucleotide identity (%)**	**Alignment length (nt)**	**Description**
Iso_Amp1	KU577908	I-31	amp	42..860	Bacillus stratosphericus DN14_7A9	+	CP007436.1	98	815	Bacillus pumilus strain MTCC B6033, complete genome
							CP009108.1	97	815	Bacillus pumilus strain GR-8, complete genom
							CP011150.1	97	815	Bacillus pumilus strain W3, complete genom
							CP012482.1	93	815	Bacillus pumilus strain NJ-V2, complete genom
							CP012329.1	93	815	Bacillus pumilus strain NJ-M2, complete genom
							AP014928.1	93	814	Bacillus pumilus DNA, complete genome, strain: TUAT
Iso_Amp2	KU577909	I-31	amp	284..1,216	Bacillus aryabhattai DN67_5C7	+	CP001983.1	99	933	Bacillus megaterium QM B1551, complete genome
							CP009920.1	96	933	Bacillus megaterium NBRC 15308 = ATCC 14581, complete genom
							CP001982.1	95	933	Bacillus megaterium DSM319, complete genom
							CP003017.1	93	933	Bacillus megaterium WSH-002, complete genom
							CP010586.	92	933	Bacillus megaterium strain Q3, complete genom
Iso_Amp3	KU577910	I-31	amp	880..1,692	Pseudovibrio ascidiaceicola DN64_8G1 & DN64_1D03	+	-	-	-	-
Iso_Chlor1	KU577911	I-31	chlor	654..1,154	Bacillus stratosphericus DN14_7A9	+	CP000813.2	96	501	Bacillus pumilus SAFR-032, complete genome
							CP009108.1	96	489	Bacillus pumilus strain GR-8, complete genom
							KF734937.1	94	501	Bacillus pumilus strain Jo2 chloramphenicol O-acetyltransferase Cat (cat
							K00544.1	93	501	B.pumilus cat-86 (chloramphenicol-acetyltransferase) gen
							CP011150.1	92	501	Bacillus pumilus strain W3, complete genom
							CP012482.1	91	501	Bacillus pumilus strain NJ-V2, complete genom
							CP012329.1	91	501	Bacillus pumilus strain NJ-M2, complete genom
							CP010075.1	91	489	Bacillus sp. WP8, complete genom
							AP014928.1	91	489	Bacillus pumilus DNA, complete genome, strain: TUAT
							CP007436.1	91	489	Bacillus pumilus strain MTCC B6033, complete genome
Iso_Gent1	KU577912	I-31	gent	2,941..3,270	Bacillus stratosphericus DN14_7A9	+	-	-	-	-
Iso_Gent1	KU577912	I-31	gent	3,399..3,845	Bacillus stratosphericus DN14_7A9	+	-	-	-	-
Iso_Ami1	KU577919	I-31	ami	884..2,065	Bacillus horikoshii DN9_1A9	-	-	-	-	-
Iso_Trim1	KU577924	I-31	trim	71..631	Flavobacteriaceae sp. DN50_6C1	+	-	-	-	-
Iso_Trim2	KU577925	I-31	trim	1..648	Nonlabens arenilitoris DN166_3E9	+	-	-	-	-
Iso_Trim3	KU577926	I-31	trim	3,032..3,562	Pseudovibrio sp. DN49_8H4	+	-	-	-	-
Iso_Trim4	KU577927	I-31	trim	254..736	Bacillus horikoshii DN9_1A9	+	-	-	-	-
Iso_Trim5	KU577928	I-31	trim	1,639..2,124	Bacillus algicola DN53_3H5	+	-	-	-	-
Iso_Trim6	KU577929	I-31	trim	1,160..1,645	Bacillus sp. DN88_4G3	-	-	-	-	-
Iso_Trim7	KU577930	I-31	trim	1,576..2,088	Acinetobacter radioresistens DN138_5C8	+	-	-	-	-
Iso_Trim8	KU577931	I-31	trim	623..841	Aquimarina megaterium DN30_1H2	-	-	-	-	-
Iso_Trim9	KU577931	I-31	trim	810..1,562	Aquimarina megaterium DN30_1H2	-	-	-	-	-
Iso_Trim10	KU577932	I-31	trim	4,902..5,537	Flavobacteriaceae sp. DN112_6A5	+	-	-	-	-
Iso_Trim11	KU577933	I-31	trim	1,718..2173	Psychrobacter celer DN193_4B9	+	-	-	-	-
Iso_Trim12	KU577934	I-31	trim	454..939	Flavobacteriaceae sp. DN105_1H3	-	-	-	-	-
Iso_Rif1	KU577935	I-31	rif	425..841	Bacillus stratosphericus DN14_7A9	-	CP011150.1	98	415	Bacillus pumilus strain W3, complete genome
							AP014928.1	97	415	Bacillus pumilus DNA, complete genome, strain: TUAT
							CP007436.1	97	415	Bacillus pumilus strain MTCC B6033, complete genom
							CP012482.1	95	415	Bacillus pumilus strain NJ-V2, complete genom
							CP012329.1	95	415	Bacillus pumilus strain NJ-M2, complete genom
							CP009108.1	95	415	Bacillus pumilus strain GR-8, complete genom
Iso_Rif2	KU577936	I-31	rif	5,293..5,709	Bacillus aryabhattai DN67_5C7	+	CP001986.1	100	417	Bacillus megaterium QM B1551 plasmid pBM300, complete sequence
							CP011051.1	96	417	Bacillus subtilis strain T30, complete genom
							CP010434.1	96	417	Bacillus subtilis subsp. spizizenii strain NRS 231, complete genom
							CP002183.1	96	417	Bacillus subtilis subsp. spizizenii str. W23, complete genom
Env_Ami1	KU577938	I-31	ami	139..531	n/a	+	-	-	-	-
Env_Trim1	KU577940	I-31	trim	199..711	n/a	+	-	-	-	-
Env_Trim2	KU577941	I-31	trim	176..664	n/a	+	-	-	-	-
Env_Trim3	KU577942	I-31	trim	398..889	n/a	+	-	-	-	-
Env_Ami2	KU577944	I-31	ami	2,700..3,374	n/a	-	-	-	-	-
